# In silico and in vitro investigations of novel Siglec-1 inhibitors in a microglial cell model

**DOI:** 10.1007/s00210-025-04962-7

**Published:** 2026-01-21

**Authors:** Shane Prenzler, Anna Lohning, Oren Cooper, Santosh Rudrawar, Thomas Haselhorst, Shailendra Anoopkumar-Dukie

**Affiliations:** 1 School of Pharmacy and Medical Sciences, Griffith University, Gold Coast, Australia; 2 Faculty of Health Sciences and Medicine, Bond University, Robina, Gold Coast 4229 Australia; 3 Institute for Biomedicine and Glycomics, Griffith University, Gold Coast, Australia

**Keywords:** Siglec-1, Sialoadhesin, CD169, SAR, ELISA, HAPI

## Abstract

The purpose of this study was to examine the differences which exist between murine, human, and rat Siglec-1, then to research novel inhibitors and assess applicability of HAPI cells to study Siglec-1. Using molecular modelling software, amino acid sequences were analysed for differences across orthologs. Compound library screening of human Siglec-1 was performed, 9 compounds of interest were identified, and 4 were included additionally out of clinical interest. Rational structure activity analysis was conducted on orthologs and chosen compounds. Competitive inhibition enzyme-linked immunosorbent assay (ELISA) using Siglec-1 was used on these 13 chosen compounds. This was followed by lipopolysaccharide (LPS) treatment of HAPI cells and Siglec-1 detection ELISA to confirm upregulation of Siglec-1. All 13 compounds were analysed using resazurin in HAPI cells with and without treatment of LPS. The N-terminal of murine Siglec-1 varies by approximately by 22% and 11% compared to human and rat orthologs respectively. Murine Siglec-1 includes Leu107 while rat and human share the more polar Ser107, despite a highly similar structure overall. Four compounds showed definable IC_50_ values. LPS stimulation compared to normal growth conditions provided significant Siglec-1 upregulation. Analysis of chosen compounds was carried out using resazurin and there was no significant cytotoxicity in Siglec-1 upregulated cells. Siglec-1 is not as highly conserved across species as previously thought, but human and rat maybe be more similar than either to mouse. HAPI cells were shown as an appropriate microglial cell model, with capable of showing upregulation of Siglec-1 under LPS conditions.

**Supplementary information:**

The online version contains supplementary material available at 10.1007/s00210-025-04962-7.

## Introduction

**Fig. 1:**
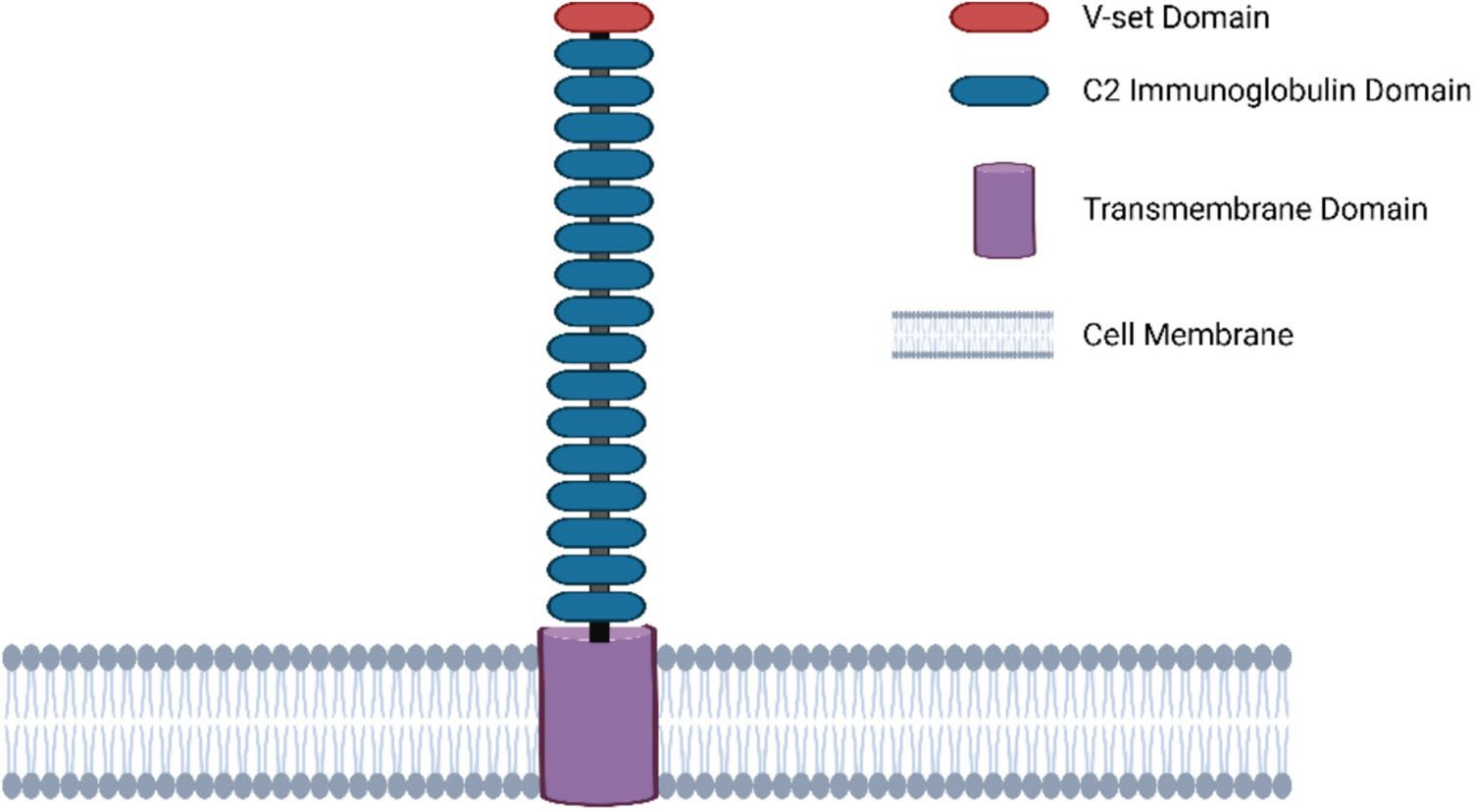
The structure of Siglec-1 (created in https://BioRender.com)

Sialic acid-binding immunoglobulin-like lectin-1 (Siglec-1), also named Sialoadhesin (CD169), constitutes the largest protein within the Siglec family of transmembrane receptors. Its extracellular region comprises 16 C2 domains and one V-set binding domain, within which the N-terminal region houses the sialic acid binding site (see Fig. 1) (Prenzler et al. 2023). Siglec receptors are classified into two subcategories based on the degree of structural conservation across mammalian species. The first category, known as highly conserved Siglec receptors, includes Siglec-1, Siglec-2, Siglec-4, and Siglec-15 (Bornhöfft et al. 2018; Prenzler et al. 2023). These receptors exhibit a high degree of structural preservation in their amino acid sequences and overall structure across different mammalian species (Bornhöfft et al. 2018). The second subcategory consists of the CD33-related Siglec receptors, which show a higher degree of variability in their amino acid sequences and structure (Bornhöfft et al. 2018). The primary role of Siglec-1 is described as a recognition receptor for sialylated pathogens, a classification supported by its lack of internal signalling motifs common to other Siglec receptors (Crocker et al. 2007).

Found primarily on innate immune cells, including dendritic cells, macrophages, and, to a lesser extent, monocytes, Siglec-1 serves numerous immune functions (Crocker et al. 2007). It is particularly important for antigen presentation and is actively upregulated during inflammation. (York et al. 2007, Xiong et al. 2009, Bao et al. 2010, Rose et al. 2013, Xiong et al. 2014; Eakin et al. 2016; Grabowska et al. 2018; Perez-Zsolt et al. 2019b; Ostendorf et al. 2021). In dendritic cells and macrophages, Siglec-1 facilitates the presentation of bound antigens directly to adaptive immune cells (e.g. T-lymphocytes), thereby initiating adaptive immune responses (Pino et al. 2015). This endocytic ability is exploited by enveloped viruses for trans-infection, notably SARS-CoV-2 and HIV-1, as well as other mammalian viruses (Sewald et al. 2015, Jobe et al. 2016, Bracq et al. 2018, Ganor et al. 2019, Perez-Zsolt et al. 2019a, b; Dupont et al. 2020; Liu et al. 2020, Perez-Zsolt et al. 2021). Beyond its role in viral entry, Siglec-1 may play a critical role in the pathology of sepsis (Sundar and Sires 2013; Wu et al. 2016). Enhanced Siglec-1 expression is hypothesised to confer immune tolerance by inducing the upregulation of TGF-β1 and subsequently reducing TNF-α expression (Sundar and Sires 2013; Wu et al. 2016).

The sialic acid-binding site and ligand affinities of murine Siglec-1 have been extensively characterised in previous studies, with less attention being given to other orthologs (May et al. 1998; Crocker et al. 1999; Zaccai et al. 2003, Bukrinsky et al. 2004, Zaccai et al. 2007, Nycholat et al. 2012). Several ligands have been developed with the goal of inhibiting the Siglec-1 sialic acid binding site and have demonstrated greater affinity for Siglec-1 than its natural ligands. Siglec-1 shows a greater preference for 2,3-linked over 2,6-linked alpha-sialosides, with further reduced affinity for 2,8-linked alpha-sialosides (Bornhöfft et al. 2018). Both 2,3-sialyllactose and 2,6-siallylacyose are both known naturally occurring ligands of Siglec-1, with the former having greater affinity (Zaccai et al. 2003). Notably, glycerol side chain modifications of the base compound methyl-α-Neu5Ac resulted in further improvement in binding affinity for Siglec-1 (Zaccai et al. 2003). When reported in terms of relative inhibitory potency (rIP) against methyl-α-Neu5Ac (rIP = 1), the modified compounds Me-α−9-N-benzoyl-amino-9-deoxy-Neu5Ac (BENZ), Me-α−9-N-(naphthyl-2-carbonyl)-amino-9-deoxy-Neu5Ac (NAP), and Me-α−9-N-(biphenyl-4-carbonyl)-amino-9-deoxy-Neu5Ac (BIP) showed greater affinity with rIP values of 2.1, 11, and 13, respectively (Zaccai et al. 2003). This binding specificity is mediated by a set of conserved amino acid residues in the Siglec-1 binding pocket, including Trp2, Tyr44, Arg97, Arg105, Trp106, Leu107, and Val109, as structurally determined in murine Siglec-1 (Bornhöfft et al. 2018). Of these, Trp2, Arg97, and Trp106 are especially critical for alpha-sialoside binding; mutagenesis studies have demonstrated that the substitution of these key residues significantly reduces Siglec-1 function (Zaccai et al. 2003). This applies in particular to Arg97, which forms an important salt bridge with the carboxylate of alpha-sialosides (May et al. 1998).

Currently, little information is known regarding the role of Siglec-1 in neuroinflammation, despite numerous studies commenting on its expression patterns within the central nervous system (CNS) (Perry et al. 1992; Groh et al. 2016; Siddiqui et al. 2019; Ostendorf et al. 2021). Notably, Siglec-1 expression on microglia was shown to be regulated by the presence of an unknown factor in serum or plasma (Siddiqui et al. 2019). As a result, it was found that microglia populations existing beyond the blood–brain barrier (BBB) do not express Siglec-1 (Siddiqui et al. 2019). However, when microglial cells are exposed to plasma or serum such as when the blood brain barrier is comprised (i.e. head injury), they then start to express Siglec-1 (Siddiqui et al. 2019). In the neurodegenerative disease ceroid lipofuscinosis (CLN), a disorder characterised by dysregulated lysosomal storage that eventually proves fatal, Siglec-1 has been shown to play an important role (Groh et al. 2016). There were two mouse models of CLN that were utilised to analyse the role of Siglec-1 in CLN, these were known as Ppt1^−/−^ and Cln3^−/−^ (Groh et al. 2016). Crossbreeding of Ppt1^−/−^ and Cln3^−/−^ with Siglec-1 deficient mice (Sn^−/−^) created Ppt1^−/−^ Sn^−/−^ and Cln3^−/−^ Sn^−/−^ models (Groh et al. 2016). In both cases, the cross bred models showed that degenerative alterations had significantly reduced (Groh et al. 2016). In Ppt1^−/−^ Sn^−/−^ mice, a substantially improved clinical phenotype was observed with a longer lifespan noted (Groh et al. 2016). Furthermore, there was a noted attenuation of M1-polarised microglia, coupled with a reduction in the expression of pro-inflammatory cytokines (Groh et al. 2016). It was also recently discovered that Siglec-1 upregulation was present in active multiple sclerosis brain lesions but not in chronic multiple sclerosis brain lesions (Ostendorf et al. 2021).

Siglec-1 is recognised as a highly conserved Siglec receptor, where the tertiary structure, function, and amino acid sequence is highly preserved between mammalian species (Bornhöfft et al. 2018). However, studies of other ‘conserved’ Siglecs have revealed that significant structural differences can still exist between species (Wöhner et al. 2012; Bornhöfft et al. 2018). A prominent example of this interspecies variability is Siglec-2 (CD22), which exhibits considerable differences between human and mouse variants. These disparities necessitated the development of the transgenic Huki murine model, which expressed human Siglec-2 so to accurately facilitate its study. (Wöhner et al. 2012). Despite the clear interspecies differences demonstrated in other Siglecs, little comparative analysis has been conducted between mammalian equivalents of Siglec-1 regarding potential differences in ligand binding affinities. Therefore, the appropriate selection of a cell line is critical. Marked interspecies differences in Siglec-1 could compromise the observation of true inhibitory effects, making species-specific study essential (Wöhner et al. 2012). Furthermore, the choice of cell line is paramount because many currently used immortalised lines may differ in phenotype compared to primary cells (Stansley et al. 2012). This is particularly relevant for microglial cells. A recent review showed that HAPI cells most closely resemble the phenotype of primary microglial cell lines and the downstream effects of interventions would be potentially more comparable (Stansley et al. 2012). Given these knowledge gaps, it is ideal to further elucidate the differences in Siglec-1 variants across species to enable more specific targeting in in vitro studies. Beyond this structural understanding, the potential role of Siglec-1 in disease states is under-explored, despite its proven involvement in innate immune cell endocytosis, transcellular signalling, possible intracellular signalling, and its active upregulation in inflammatory conditions (Delrue et al. 2010; van Dinther et al. 2018; van Dinther et al. 2019; Liu et al. 2020). Therefore, acquiring sufficient information now to facilitate its future therapeutic targeting will be essential.

Screening of existing compound libraries is a widely adopted tool in modern drug discovery that has not yet been applied to Siglec-1 (Aaftaab et al. 2019). Where possible, the repurposing of existing drugs has become a favourable strategy, offering advantages over traditional drug discovery, a fact made especially evident during the COVID-19 pandemic (Aaftaab et al. 2019; Gurung et al. 2021). The benefits of drug repurposing include significant reductions in development time, procurement cost, financial investment, and risk of failure. Furthermore, the clinical safety and efficacy data for these compounds are generally well-documented (Aaftaab et al. 2019).

The primary aims of this study were to establish the structural and functional differences that exist between Siglec-1 orthologs (across mouse, human, and rat) and to determine the impact of these differences on the binding affinities of chosen ligands. A secondary objective was to appraise the use of rat-derived HAPI cells as an acceptable microglial model for Siglec-1 studies and as a possible alternative to human microglia in vitro. To achieve these goals, potential ligands were first selected in silico based on existing compound library screens on human Siglec-1 or out of clinical interest. These ligands are then collectively referred to hereafter as the chosen ligands. Differences that exist between the mouse, human, and rat Siglec-1 N-terminal regions were primarily investigated computationally, as the published mouse X-ray crystal structure (1QFP) has rarely been compared with other orthologs (Florea et al. 2005). Both molecular docking and Rational Structure–Activity Relationship (RSAR) analyses were utilised across the N-terminal regions of all three orthologs to compare protein binding and ligand affinity. When discussing Siglec-1 in this article, the N-terminal region is the focus, unless otherwise specified.

**Fig. 2:**
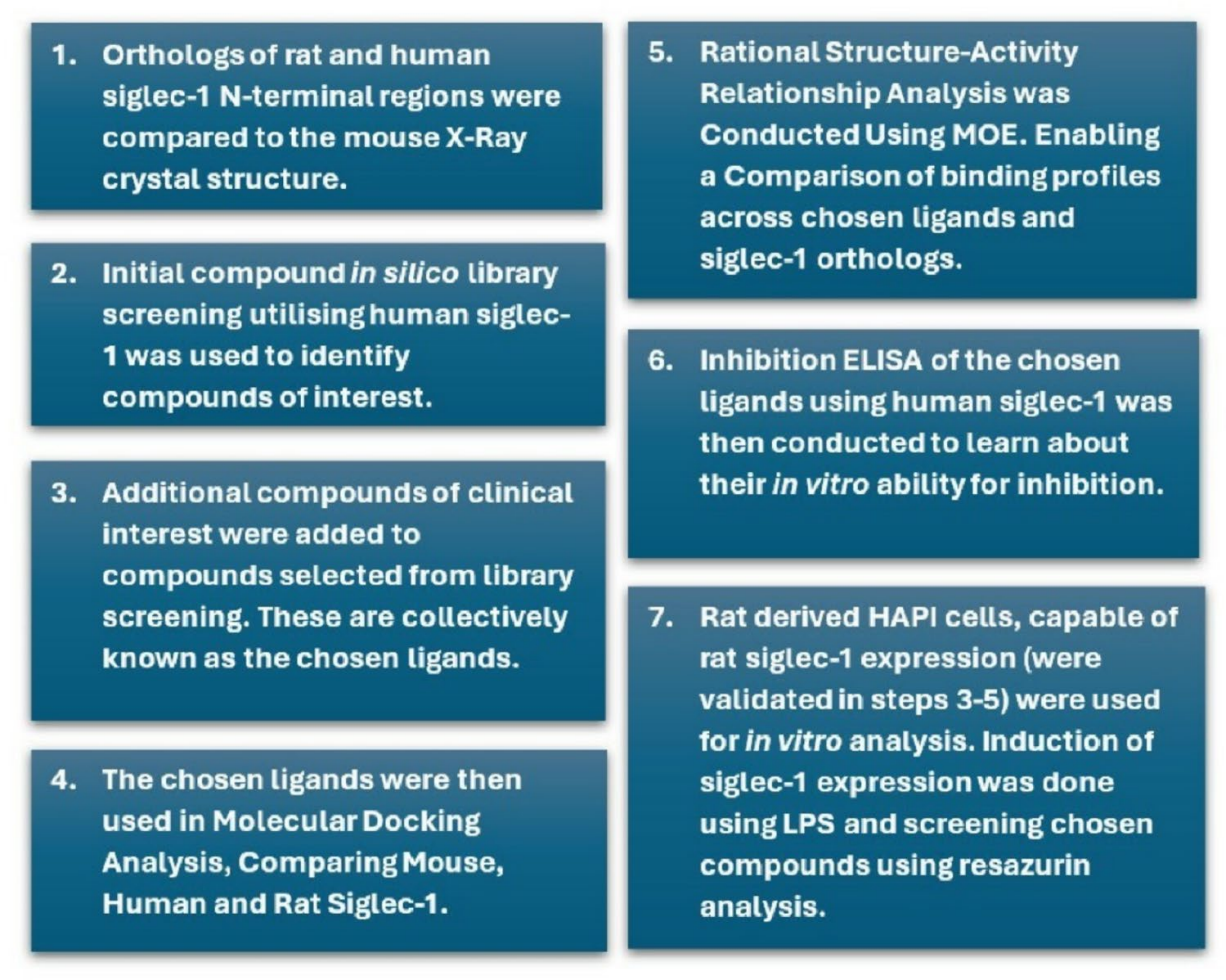
An overview of the study design in sequence including the methods used

Following the computational work, the study transitioned to in vitro methods (see Fig. 2). A preliminary screening of the chosen ligands was conducted using ELISA techniques and human Siglec-1 to validate earlier in silico findings (Bock and Kelm 2006; Koliwer-Brandl et al. 2011). Crucially, an assessment of HAPI cells was conducted to validate their use as an appropriate model for Siglec-1 studies, replacing primary cell lines. This involved combining the earlier in silico assessments demonstrating the structural similarity between rat Siglec-1 and human Siglec-1, then by verifying that HAPI cells express Siglec-1 that is inducible under LPS stimulation (a critical feature of microglial cells in vivo) (Stansley et al. 2012; Siddiqui et al. 2019). Finally, to ensure the reliability of future intervention studies, the cytotoxic effects of the chosen ligands on HAPI cells (with and without LPS induction) were determined using resazurin assays (Anoopkumar-Dukie et al. 2020).

## Methods

### Structural assembly of Siglec-1 orthologs and molecular docking

The N-terminal domains of human Siglec-1 (UniProt ID: Q9BZZ2.2) and rat Siglec-1 (UniProt ID: A6HQC1) were assembled via SwissModel homology modelling. The FASTA files were downloaded from UniProt, and the tertiary structures were developed using the X-ray crystal structure of mouse Siglec-1 (PDB: 1QFP) as the template (Florea et al. 2005). The sequence alignment comparison of PDB structures for mouse, human and rat Siglec-1 was completed using Chimera-X. This was completed by overlapping the N-terminal domains of Siglec-1 across both human and rat with that of 1QFP (May et al. 1998; Hartnell et al. 2001; Pettersen et al. 2004; Florea et al. 2005; Waterhouse et al. 2018).

Following this, 22,843 compounds from various publicly accessible libraries, originally available as 2D SMILES formats were chosen for screening. Three-dimensional structures were generated using DataWarrior, and duplicate entries were removed prior to downstream analysis (Sander et al. 2015). Both AutoDockVina and YASARA were then utilised for analysis on the N-terminal domain of human Siglec-1. This allowed for comparison between binding affinity and molecular interactions of each ligand and the binding site of Siglec-1 (Trott and Olson 2010; Land and Humble 2018, Eberhardt et al. 2021). The newly formed human Siglec-1 N-terminal domain was then prepared by deleting water molecules, then adding both polar hydrogens and Kollman charges (Trott and Olson 2010; Land and Humble 2018, Eberhardt et al. 2021). A grid box was formed around binding site significant amino acids (ie. Trp2, Tyr44, Arg97, Arg105, Trp106, Ser107 and Val109) at x-20 Å, y-20 Å and z-24 Å centres. Ligands were selected based on their potential to interact with these amino acids, particularly Arg97, as previous studies have concluded that its substitution would abrogate Siglec activity (May et al. 1998). After determining a perimeter for binding, high affinity ligands were chosen from screened compound libraries (see Appendix Table 1). In addition to these compounds, 2,3-sialyllactoce, 2,6-sialyllactose, methotrexate, tamsulosin, and prazosin were chosen out of clinical interest but were not included in initial in silico screening (see Appendix Table 1).

Lipophilicity mapping was carried out to compare the differences that exist between each ortholog of Siglec-1. A flexible docking procedure using the known ligand Me-α−9-*N*-benzoyl-amino-9-deoxy-Neu5Ac (BENZ) was carried out with each ortholog of Siglec-1 (mouse, human, and rat). This was to emphasise the location of the binding site in rendered images and to illustrate the potential binding site changes due to polymorphic changes present (May et al. 1998; Zaccai et al. 2003). Surface rendered lipophilicity and interactions were captured using AutoDockTools/AutoDockVina, Chimera, and Discovery Studio (Pettersen et al. 2004; Trott and Olson 2010; Biovia et al. 2016). The molecular docking procedure required that the 1QFP structure of murine Siglec-1, as well as the human and rat homology models be docked with BENZ. AutoDockTools was used to process each macromolecule and BENZ, where solvent was removed, non-polar hydrogens were merged and charges added (Kollman charges for orthologs and Gasteiger charges for BENZ) (Trott and Olson 2010). After this, a grid box of (*x*−20 Å, *y*−20 Å, *z*−20 Å) was placed around Arg97 to tether the docking to this essential amino acid. AutoDockVina was then used to generate a list of docking scores and conformations (Trott and Olson 2010). Preserving known molecular interactions between the ligand and the Siglec-1 binding site was given priority, as were the known intramolecular interactions of the bound ligand BENZ (Zaccai et al. 2003; Frank et al. 2023). A semi-flexible docking procedure was carried out using AutoDockTools on each Siglec-1 ortholog and BENZ was granted reduced torsions during the docking procedure. The bonds between carbons C6-C8 were made inflexible to maintain the important 8O-hydroxyl C1 carboxylate hydrogen bond known to sialosides (Frank et al. 2023). The flexibility of binding pocket amino acids was included to simulate a more accurate molecular environment within the binding pocket of Siglec-1 for docking of BENZ. This included flexibility of amino acids Trp2, Tyr44, Arg97, Arg105, Trp106, Leu107 (Ser107 in human and rat Siglec-1), and Val109 (May et al. 1998; Zaccai et al. 2003). Conducting semi-flexible docking was found to preserve necessary interactions and enable adequate illustration of the Siglec-1 sialic acid binding site (Abreu et al. 2012). Along with allowing selected Siglec-1 binding site amino acids flexibility, torsions in BENZ were allowed except for those between carbons C6, C7, and C8 (Abreu et al. 2012; Frank et al. 2023). Keeping the BENZ ligand partially flexible allowed for the 8O hydroxyl to C1 carboxylate hydrogen bond to be maintained as an intramolecular interaction after extracting the BENZ ligand from the X-ray crystal structure of 1OD9 (BENZ bound to murine Siglec-1) (Zaccai et al. 2003; Frank et al. 2023). This interaction is an important and biologically vital intramolecular interaction to stabilise sialosides and enables adequate presentation of the C1 carboxylate of BENZ to ARG97 for salt bridge formation (Frank et al. 2023).

### Rational structure–activity analysis

RSAR is a comparative quantitative analysis which was employed to analyse the binding sites across mouse, human and rat Siglec-1 (Temml and Kutil 2021). This was to further understand how the alterations in mutations to the Siglec-1 binding site which exist across species may affect the binding profiles of compounds of interest (Gurung et al. 2021; Temml and Kutil 2021; Lévêque et al. 2022; Mamada et al. 2022). To perform a quantitative structure–activity relationship (QSAR), a larger amount of biological data is usually required, which is missing for Siglec-1 and was therefore not possible in this study (Temml and Kutil 2021). To investigate the structure–activity relationships that are important for our compounds of interest the limitations of having fewer biological data were addressed by the following approaches:

- Compositional differences within the Siglec-1 binding sites provided a rational approach to direct selection of molecular descriptors. The most significant impact of these differences manifested in differences in lipophilicity and shape of the binding site and thus descriptors based on these characteristics were selected.
- Predicted binding interaction data, generated from molecular docking studies, were used as surrogate biological data for the structure–activity analysis.
- Training set included known binders such as sialic acid, Prop5Ac, BPC-Neu5Ac, BENZ, 2,6-SL, 2,3-SL along with decoys (positive and negative controls respectively). Test set compounds were drawn from other high-scoring compounds, identified in the YASARA screening (velpatasvir, cefpiramide, irinotecan, and nilotinib) along with a range of structurally diverse compounds.
- The potential for over-fitting the data was minimised by employing a robust statistical analysis involving multiple linear regression with further cross validation (Duchowicz 2018; Tukur et al. 2019).
- Model validation was performed by including a set of structurally diverse compounds to probe the physical and chemical landscape (Duchowicz 2018; Tukur et al. 2019; Temml and Kutil 2021).

RSAR was performed using Molecular Operating Environment (MOE), whose use in quantitative studies of structure–activity relationships has already been documented (Lakhlili and Ibrahimi 2016). The RSAR model was based on known ligands that show binding to Siglec-1, plus one negative decoy, while descriptors were used for the purpose of model fields. This list of compounds constituted the training set of compounds and was used to develop a multivariable linear regression model (MLRM); this was later applied to the test set of compounds to see how well they correspond with this developed MLRM (Duchowicz 2018; Lévêque et al. 2022). MLRM links between molecular descriptors and biological activity of ligands (Duchowicz 2018). This can be represented by the following equation:

1 $$\mathrm S={\mathrm M}_1{\mathrm X}_1+{\mathrm M}_2{\mathrm X}_2+{\mathrm M}_3{\mathrm X}_3+{\mathrm M}_4{\mathrm X}_4+...\mathrm C$$

where *S* stands for the dependent variable representing biological activity, *X* for the independent variable (descriptor), *M* for the regression coefficients of the various independent variables (descriptors), and *C* for the regression constant (Ul-Haq et al. 2016).

Using MOE, it was possible to conduct RSAR analysis across mouse, human, and rat Siglec-1 N-terminals to assess the theoretical binding affinities of compounds interest. The training set of compounds used to conduct RSAR and to develop the linear model regression. This linear regression model was then utilised for the test set of compounds that are shown in the below table (see Table 1). Importantly, the negative control used in the training set (Decoy1_mod) was obtained from the DUD-e database (http://dude.docking.org) (Mysinger et al. 2012). Although the DUD-e database contains decoys useful for many targets, none are specifically for Siglec receptors. The closest transmembrane protein was selected in place of Siglec-1 to obtain a decoy from this database. The chosen transmembrane receptor was a viral glycoprotein adhesion protein since they frequently bind sialic acid (Hammonds et al. 2017). To accommodate for the lack of data regarding the suitability of usable decoys, other compounds that were structurally diverse were selected for inclusion. These structurally diverse compounds included glycyrrhetinic acid (GA), 6-(methylsulfinyl)hexyl isothiocyanate (6MITC), cortisol, and lutein, none of which were expected to bind. As these compounds were not expected to bind to the sialic acid binding site of Siglec-1 in the test set, they were useful to further assess the competency of the developed model. In addition, the test included compounds that had shown binding ability or were of clinical interest.

**Table 1:** Training set compounds vs. test set compounds for RSAR analysis

Training set compounds	Test set compounds
2-Phenyl- Prop5Ac (Prop5Ac)	Prazosin
Me- α −9-N-(biphenyl-4-carbonyl)-amino-9-deoxy-Neu5Ac (i.e. BPC-Neu5Ac)	Tamsulosin
Me-α−9-*N*-benzoyl-amino-9-deoxy-Neu5Ac (i.e. BENZ)	Methotrexate
Velpatasvir	STK545231
2,6-Sialyllactose (2,6-SL)	STK516011
2,3-Sialyllactose (2,3-SL)	STK548837
Cefpiramide	STK882436
Nilotinib	Glycyrrhetinic acid (GA)
Irinotecan	6-(methylsulfinyl)hexyl isothiocyanate (6MITC) ^*^
Decoy1_mod (negative control)	Cortisol ^Ѱ^
Sialic acid	Lutein^*^

*These compounds were added out of professional interest to the test set but were not included in any other analysis other than RSAR and in silico screening

ѰCortisol was added as a potential negative control in the test set but was not included in any other analysis other than RSAR and in silico screening. The use of cortisol as a potential negative control came after many of the binding site amino acids of Siglec-1 were seen to be polar

### Enzyme linked immunosorbent assay for Siglec-1

The ELISA for detecting Siglec-1 inhibition was based on methods previously described by Koliwer-Brandl et al. (Bock and Kelm 2006; Koliwer-Brandl et al. 2011). A standard curve was prepared using 4 µg/mL fetuin (Sigma-Aldrich F2379) and asialofetuin (Sigma-Aldrich A4781) solutions in NaHCO_3_ (pH 9.7), which represented 100% and 0% Siglec-1 binding, respectively. These stock solutions were mixed to generate standards ranging from 10 to 90% binding. A 10 µL aliquot of each standard was added to the wells of an OptiPlate-384 High Bind Black Microplate (Perkin Elmer catalogue no. 6005520). The plate was centrifuged (2000 rpm for 2 min at 4 °C), sealed with parafilm and incubated overnight at 2–8 °C. The following day, human Siglec-1 (R&D Systems, cat. no. 5197-SL) was diluted to a working concentration of 5 µg/mL in 1xTBS. Anti-human IgG-AP (ThermoFisher catalogue no. A18820) was then complexed with the Siglec-1 working solution at a 1:500 ratio (1 µL of IgG per 500 µL Siglec-1 solution) and allowed to rest for 5 min at 2–8 °C. Inhibitors being tested were diluted separately in 1xTBS. The microplate was removed from the refrigerator and washed 5 times with 20 µL of TBS-Tween per well, with gentle tapping onto tissue paper between each wash. The microplate was then treated with 10µL of the IgG-AP/Siglec-1 complex to each well, followed by centrifugation (2000 rpm at 4 °C). The plate was again sealed with parafilm and incubated for a further 4 h at 2–8 °C. Following incubation, the plate was washed four times with 20 µL TBS-Tween per well and once with 1xTBS, again with intermediate tapping onto tissue paper. Immediately, 20 µL of 20µM fluorescein diphosphate (FDP) (Invitrogen catalogue no. F2999) diluted in alkaline phosphatase (AP) buffer solution was added to each well. Fluorescence was measured immediately using an excitation wavelength of 485 nm and emission of 535 nm.

### Induction and quantification of soluble Siglec-1 in HAPI cell supernatant

The successful induction of Siglec-1 expression in HAPI cells following LPS treatment was confirmed by quantifying soluble Siglec-1 (sSiglec-1) in the supernatant using an ELISA kit (MyBiosource, cat: MBS049945). This validated the HAPI cell line as a suitable in vitro model for studying Siglec-1 function under inflammatory conditions by LPS*.* The ELISA kit provided a detectable range of 3.12 to 100 ng/mL for Siglec-1 with a sensitivity of 1.0 ng/mL*.* The kit was stored at 2–8 °C. All standards, samples, and blanks were run in triplicate for statistical analysis. Twenty-four hours after treatment with 10 ng/mL LPS, cell supernatants were collected and centrifuged at 3000 rpm for 20 min. HAPI cells that received only media served as the control; cell media was also used to run a standard curve to confirm it did not contribute to detected absorbance (Forrester et al. 2018). First, 50 µL of standard, sample and buffer are added to the corresponding wells (i.e. standard wells, sample wells, and blank wells). Then, 100 µL of HRP-conjugate reagent is added to each well. At this point, the plate is covered with the closure plate membrane and allowed to incubate for 60 min at 37 °C. Following incubation, a wash step was performed four times using 300 µL of a 1:20 dilution of wash buffer per well, followed by inverting the plate and gently tapping it onto paper towels to facilitate fluid detachment. Next 50 µL of chromogen A solution was added to each well, immediately followed by 50 µL of chromogen B solution. It is important to protect the plate from light after the addition of chromogen B. The plate was protected from light and incubated for 15 min at 37 °C before the addition of the stop solution. The absorbance (Optical Density, O.D.) was measured at 450 nm.

### Resazurin assay of chosen ligands

The Resazurin assay was used to assess cell viability in the presence of candidate ligands, with and without LPS pre-treatment of cells. Resazurin (Sigma-Aldrich R7017) was prepared as a 440 µM stock solution and diluted in low-glucose Dulbecco’s Modified Eagle Medium (DMEM) (Gibco cat. no. 12320032) to form a 44 µM working solution (Anoopkumar-Dukie et al. 2020; Tseng et al. 2020). Both stock and working solutions were then stored under refrigerated conditions (2–8 °C) prior to use. Each compound concentration was performed as *n* = 9 in both normal complete cell media and lipopolysaccharide (LPS) O55:B5 (Sigma-Aldrich catalogue no. L2880). Triplicates were each performed on different plates from a different passage of cells to ensure independent replicates for conditions tested. The first day of the Resazurin assay required seeding HAPI cells at 40,000cells/well in 24-well plates by adding 100 µL of a 400,000 cells/mL suspension, followed by 220 µL of complete cell media. On the second day of the resazurin assay, an additional 40 µL of either complete cell medium or LPS 100 ng/mL is added to their respective wells. LPS 100 ng/mL becomes is diluted in complete cell medium and achieves an in-well concentration of 10 ng/mL. Additionally, 40 µL of compound concentration (diluted in complete cell media) required for treatment was also added to each applicable well. The required concentration of the compound had to be prepared as a working solution before inoculating the wells. The working solution had to take into account the dilution of the compound as soon as it was added to the well and therefore had to be 10 times more concentrated than the concentration to be tested. On day three, supernatant was removed and replaced with 400 µL of 44 µM resazurin working solution, then incubated for 3 h in the dark at 37 °C and 5% CO_2_. Fluorescence was then measured at an excitation of 535 nm and an emission of 590 nm.

## Results

### Comparative ortholog binding site analysis

**Fig. 3:**
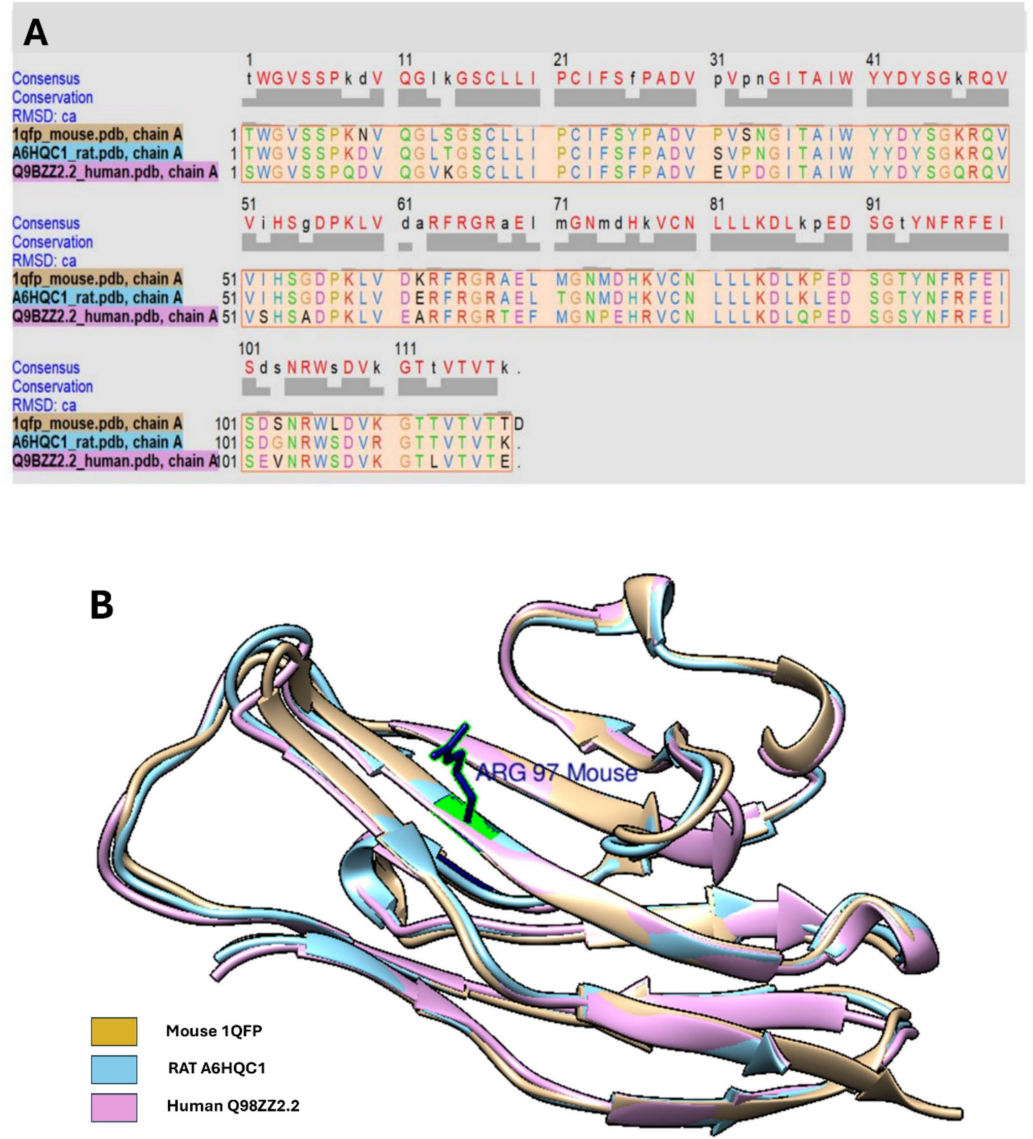
**a**, **b** Sequence alignment (**a**) and superimposition of mouse X-ray crystal structure to human and rat Siglec-1 homology models (**b**) (Pettersen et al. 2004)

Comparative binding site analysis began with retrieving the mouse Siglec-1 X-ray crystal structure from RCSB PDB (code: 1qfp), and human (geneID: Q9BZZ2.2) and rat (geneID: A6HQC1) sequences from Uniprot (May et al. 1998; Hartnell et al. 2001; Florea et al. 2005). Homology models for the N-terminal domains of the human and rat orthologs were generated using SwissModel, with the 1qfp mouse structure as the template (May et al. 1998; Waterhouse et al. 2018). Chimera was then used to compare the differences that may take place in the respective amino acid chains, and it was noted that in the sequence alignment of the N-terminal domains of the three orthologs, there was some variation observed (see Fig. 3a) (Pettersen et al. 2004). Between the 1qfp structure of mouse Siglec-1 and those generated for human and rat, there was an approximate variation of 22% and 11% in amino acid sequence, respectively. Comparatively between human and rat N-terminals, there was a noted approximate 21% variation in amino acid sequence. In contrast to the sequence variability, the structural alignment of the orthologs was highly conserved (see Fig. 3b). The root mean square deviation (RMSD) values were shown to be 0.50 Å and 0.54 Å for mouse to human and mouse to rat comparisons, respectively. The amino acid Arg97 of mouse Siglec-1 is highlighted in the superimposed structure to aid in visualisation of the sialic acid binding site (see Fig. 3b). This location should be near identical across each ortholog.

**Fig. 4:**
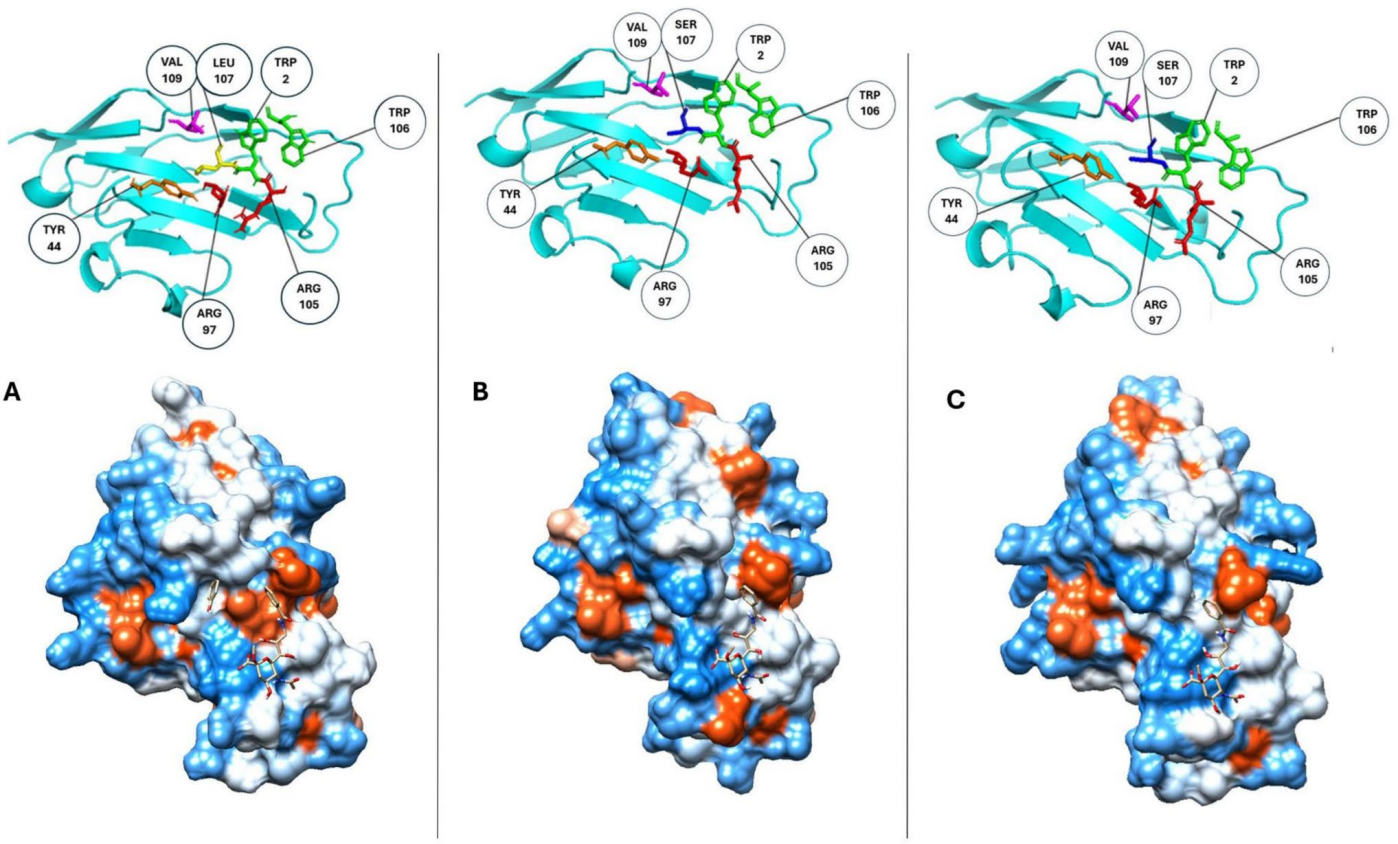
**a**–**c** Binding site comparison of murine (**a**), rat (**b**), and human (**c**) Siglec-1 N-terminal regions (Pettersen et al. 2004)

**Fig. 5:**
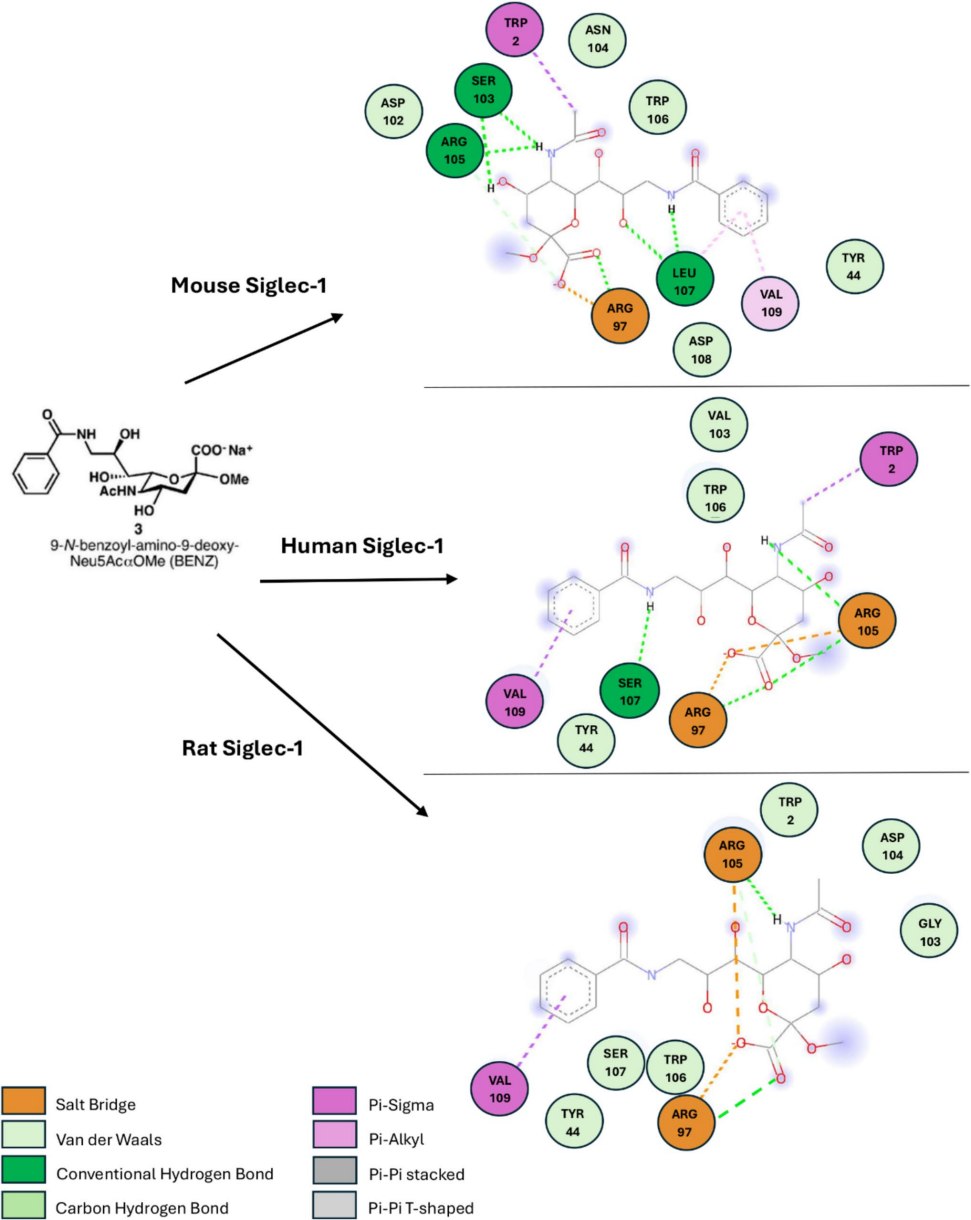
Structure activity relationships of BENZ interacting with the sialic acid binding site of Siglec-1 orthologs of mouse, human and rat (Biovia et al. 2016)

Despite the notable differences in overall N-terminal amino acid sequence, the high degree of structural conservation ensures that the binding site remains highly similar, with the only amino acid substitution being Leu107 which is consistent in both human and mouse being changed to Ser107 (see Fig. 4). To illustrate and aid in visualisation, surface mapping of hydrophilicity and lipophilicity was done to each ortholog whilst interacting with the known ligand BENZ at the sialic acid binding site (see Fig. 4) (Zaccai et al. 2003). Comparison of the docked murine Siglec-1 and BENZ complex after flexible docking and that of the X-ray crystal structure (1OD9) showed an RMSD value of 1.62 Å using PyMOL, an acceptable value for flexible docking (Williams and Kalyaanamoorthy 2021; Vittorio et al. 2024). In the lipophilic surface rendered images, blue indicates a region is hydrophilic, red is lipophilic, and white regions are neutral. It was noticed that the binding site of mouse Siglec-1 had more noticeable lipophilicity in comparison to both human and rat, and this was a consequence to Ser107 being a more hydrophilic amino acid. Using Chimera ®, the N-terminals of mouse, rat, and human (Fig. 4a, b, and c respectively) showed remarkable structural similarities, although surface rendering for lipophilicity showed some difference between binding site regions of each form of Siglec-1 in Fig. 4, likely to be in part due to the polymorphisms already identified. This change in amino acid make up within the binding site resulted in possible altered binding site and ligand molecular interactions, being evident across orthologs (see Fig. 5).

Some theoretical differences in binding site interactions of interest were observed across Siglec-1 orthologs. Despite the molecular docking procedure being identical for all three orthologs, differences were seen mainly because of additional distal polymorphisms outside of the binding pocket (see Figs. 4 and 5). All orthologs of Siglec-1 examined in this study were shown to form the critical salt bridge with Arg97 with BENZ; however, BENZ was observed to have additional hydrogen bonding and electrostatic interactions with another local Arginine residue in human and rat Siglec-1. This was seen with the theoretical interaction with Arg105, which is not an observed interaction for murine Siglec-1 and BENZ (see Fig. 5) (Zaccai et al. 2003; Trott and Olson 2010). Leu107 in murine Siglec-1 was shown to have additional hydrophobic interaction with the benzene ring of BENZ, this was absent from human and rat Siglec-1 with the more polar Ser107. Auxiliary binding site amino acid Val109 contributed pi-alkyl hydrophobic interactions with the BENZ ligand in murine Siglec-1 but was found to be a pi-sigma interaction in both human and rat Siglec-1. Another contributing polymorphic difference across all three Siglec-1 orthologs includes those at the amino acid position 103 of the N-terminal region. In this instance, each ortholog had a different polymorphism in this position with Ser103, Val103 and Gly103 having differing auxiliary interactions in murine, human and rat Siglec-1, respectively. Ser103 contributes to hydrogen bonding interactions in murine Siglec-1, while both Val103 and Gly103 contribute to hydrophobic interactions at different points (see Fig. 5). Interactions such as these help change the makeup of each ortholog binding site and even when human and rat Siglec-1 both contain the Ser107 polymorphism mean that potentially there are alterations to how a ligand will interaction in both orthologs. BENZ forms hydrogen bonding with Ser107 in human Siglec-1 but contributes hydrophobic interactions with rat Siglec-1. Meanwhile, Trp2 contributes pi-sigma interactions with mouse and human Siglec-1 and Van der Waal interactions only with rat Siglec-1. Overall, the binding affinity across each complex for the compound BENZ was shown to be similar at − 6.2kcal/mol (mouse), − 5.8kcal/mol (human), and − 6.1kcal/mol (rat). BENZ was also found to adopt the chair conformation which existed in the 1OD9 structure and is lower energy compared to other possible conformations such as the boat conformation for sialosides (Schauer and Kamerling 1997; Zaccai et al. 2003). This allowed for all noted molecular interactions between BENZ and Siglec-1 to be maintained for murine Siglec-1, these matched those seen in the 1OD9 X-ray crystal structure (Zaccai et al. 2003). Both human and rat Siglec-1 showed similar binding profiles to mouse Siglec-1 overall.

### In silico compound library screening of human Siglec-1

The in silico screen began with a compound library search using YASARA (see Table 2) (Land and Humble 2018). This was then supplemented with drugs of clinical relevance: 2,3- and 2,6-sialyllactose, methotrexate, tamsulosin, and prazosin. Both 2,3- and 2,6-sialyllactose constitute natural ligands to Siglec-1 and were chosen for the confidence they retain as positive binding controls (Prenzler et al. 2023). Methotrexate was chosen due to the role it currently has in treating relevant neoplasms and autoimmune disease, but also the expanding research that shows it has the potential to influence the neuroinflammatory environment (Koźmiński et al. 2020). The inclusion of α1-adrenoreceptor (AR) antagonists was due to the acceptance that AR antagonists have a role within the CNS that is evolvingly with our understanding (Zhang et al. 2012; Perez 2020; Latvala et al. 2022). Further molecular docking and dynamics analysis was conducted using computational docking software MOE™ (see Table 3.) (ULC 2024). Comparison of the compounds chosen for MOE in silico analysis as well as background information on why each compound was selected and any current use can be found in Table 3. Included are also the results of Siglec-1 ELISA inhibition studies and the level of inhibition observed. These compounds were then screened using a formulated Siglec-1 inhibition ELISA using human Siglec-1. The comparison between the binding affinities for Siglec-1 in the context of comparing in silico rat Siglec-1 binding affinities to that of experimental affinities in human Siglec-1 highlighted some similarities. All compounds where IC_50_ values were later found using the direct Siglec-1 (human) ELISA had theoretical affinities that were comparable across both human and rat Siglec-1. To further analyse the small trends that exist across orthologs of Siglec-1, RSAR analysis was conducted to further elaborate binding site differences.

**Table 2:** Screened compound list obtained from compound libraries

Library screened compound	*K*_d_ value (nM)
STK545231	791
STK516011	880
STK882436	385
STK548837	556
Irinotecan	1581
Nilotinib	1856
Cefpiramide	5110
Velpatasvir	513

**Table 3:** Analysis summary for chosen compounds

Name of compound	MOE molecular docking analysis			Siglec-1 inhibition ELISA*		Background of compound		
	Affinity mouse Siglec-1 (kcal/mol)	Affinity human Siglec-1 (kcal/mol)	Affinity rat Siglec-1 (kcal/mol)	IC_50_ value found using ELISA	Observed level of inhibition/(IC_50_) from ELISA	Clinical applications	Mechanism of action	Selection
**STK545231**	− 7.03	− 6.53	− 6.31	**Yes**	**(IC**_**50**_: 497.8µM) ^*^	N/A	N/A	Compound library
STK516011	− 5.94	− 6.06	− 6.73	No	No inhibition found	N/A	N/A	Compound library
STK882436	− 7.29	− 6.12	− 6.41	No	No inhibition found	N/A	N/A	Compound library
STK548837	− 6.79	− 7.23	− 6.95	No	13.91% inhibition at 1000 µM	N/A	N/A	Compound library
**2,3-Sialyllactose**	− 6.35	− 6.31	− 6.48	**Yes**	**(IC**_**50**_:71.78µM) ^*^	Natural ligand of Siglec-1 (Bornhöfft et al. 2018; Prenzler et al. 2023)	Oligosaccharide found in breastmilk (Harris et al. 2020)	Clinical interest
**2,6-Sialyllactose**	− 6.56	− 6.21	− 6.60	**Yes**	**(IC**_**50**_:149.3µM) ^*^	Natural ligand of Siglec-1 (Bornhöfft et al. 2018; Prenzler et al. 2023)	Oligosaccharide found in breastmilk (Sodhi et al. 2021)	Clinical interest
Methotrexate	− 6.79	− 6.47	− 6.63	No	No inhibition found	DMARD, chemotherapeutic agent, immunosuppressant (Hamed et al. 2022)	Tetrahydrofolate reductase inhibitor (Hamed et al.^2022^)	Clinical interest
Irinotecan	− 6.57	− 7.30	− 6.85	No	9.44% at 1000 µM	Chemotherapeutic agent (Bailly 2019)	DNA topoisomerase I inhibitor (Bailly 2019)	Compound library
Nilotinib	− 7.01	− 6.57	− 6.62	No	10.33% at 1000 µM	Chemotherapeutic agent (Kuo et al. 2018)	Tyrosine kinase inhibitor (Kuo et al. 2018)	Compound library
Cefpiramide	− 7.87	− 7.13	− 6.78	No	27.32% inhibition at 1000 µM	Broad spectrum cephalosporin (Wang et al. 2000)	Cephalosporin antibiotic, transpeptidase inhibitor (Wang et al. 2000)	Compound library
**Prazosin**	− 5.82	− 5.64	− 5.41	**Yes**	**(IC**_**50**_:586.3µM) ^*^	Anti-hypertensive, benign prostatic hyperplasia, PTSD-associated nightmares, Raynaud’s syndrome (Brogden et al. 1977; Reist et al. 2021)	Non-selective alpha1-antagonist (Brogden et al. 1977; Reist et al. 2021)	Clinical interest
Tamsulosin	− 6.26	− 5.76	− 5.69	No	19.21% inhibition at 500 µM	Benign prostatic hyperplasia (Dunn et al. 2002)	Selective alpha1-antagonist (Dunn et al. 2002)	Clinical interest
Velpatasvir	− 5.52	− 5.97	− 5.52	No	42.33% inhibition at 1000 µM	Chronic hepatitis C (Greig 2016)	NS5A inhibitor (Greig 2016)	Compound library

*IC50 values and level of inhibition obtained are only in the context of human Siglec-1 due to the inhibition ELISA utilising human Siglec-1

Compounds obtained from compound libraries with their corresponding dissociation constants (*K*_d_).

### RSAR analysis

Descriptors may influence the biological activity in a positive or negative manner and to varying extents (Danishuddin and Khan 2016). This is reflected in the multiple linear regression equation by the magnitude and sign of M where a positive value increases activity and negative values decrease it (Inc. 2008). Descriptor analysis using RSAR, is represented in Table 4, where values between and equal to − 1 and + 1 are the absolute minimum and maximum values possible. Negative values are indicative of an unfavourable relationship for binding affinity to the binding site, whereas positive values indicate a favourable relationship, with negative in this sense indicating a negative association or poorer binding affinity which contrasts with those that are positive (Inc. 2008). The value of the contribution is relative to the value of the number awarded to a given descriptor, such as + 1 would indicate the strongest association towards binding but + 0.5 would indicate a positive but less substantial association (Inc. 2008).

**Table 4:**
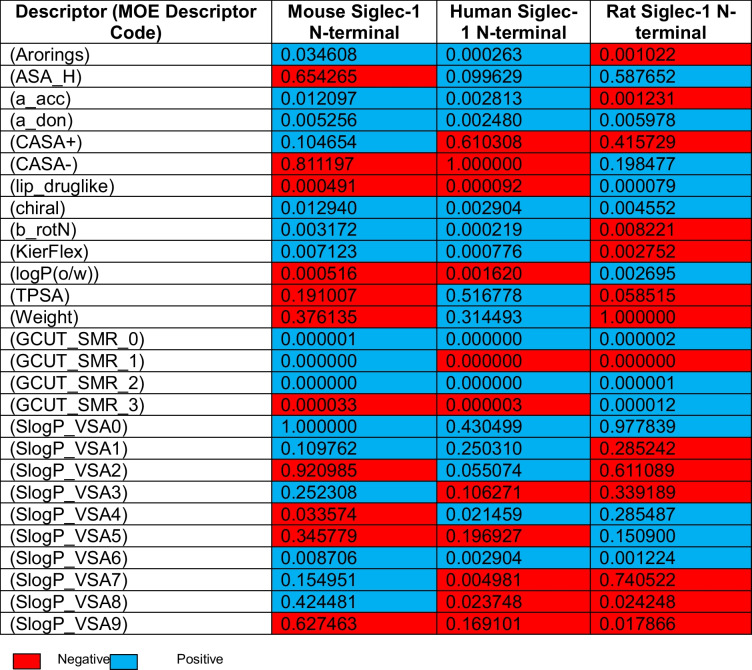
RSAR descriptor analysis of each Siglec-1 ortholog studied

Arorings is a descriptor that analyses the impact of ligand aromatic rings on binding affinity to the binding site of Siglec-1 (see Table 4) (Inc. 2008). Due to the small influence this descriptor has had on binding across Siglec-1 variants examined in this study, aromatic rings seem to be less beneficial for affinity across the forms of Siglec-1 examined. This could also be said for a_acc and a_don which relate to hydrogen bond accepting and donating groups, respectively, which include hydroxyl groups but not including acidic or basic groups, respectively (Inc. 2008). There was a noticeable difference seen for ASA_H which related to the water accessible surface area of hydrophobic atoms in the binding site. This property seemed to be relatively unimportant for human Siglec-1 but had a moderately negative association for mouse Siglec-1 yet a moderately positive association for rat Siglec-1. With regard to the charge per weighted surface area, a negative charge or CASA- was strongly associated with a negative association for binding in both human and mouse Siglec-1 but was minorly associated with a positive binding potential for rat Siglec-1 (Inc. 2008). Positively charged molecules per weighted surface area or CASA + was associated with a moderately negative association of binding to both human and rat Siglec-1 (Inc. 2008). When considering ligands that adhere to the Lipinski drug like criteria, the descriptor lip_druglike showed no negligible associations among all variants (Inc. 2008). Chirality, number of rotatable bonds, molecular flexibility, and log of octanol/water partition coefficient all also seemed to not heavily influence ligand affinity (Inc. 2008). TPSA which is a descriptor for the polar surface area of ligands showed a moderate positive association for human Siglec-1 and a slight negative association for mouse Siglec-1, but a negligible association for rat Siglec-1 (Inc. 2008). The descriptor Weight, which is a descriptor for molecular weight, showed that compounds of a large molecular weight would have a very large negative impact on binding for rat Siglec-1, a small negative impact on mouse Siglec-1 and a small positive impact or association for human Siglec-1. An example of where this might be shown would be with velpatasvir as it is the largest molecule screened by weight and had the lowest potential binding affinity across all orthologs of any compound screened, apart from prazosin (see Table 3 and Appendix Table 1). The highest level of potential affinity velpatasvir obtained was in human Siglec-1 where higher molecular weight was shown to have a small positive effect on binding. However, caveats exist whereby some compounds examined had molecular weights only slightly below that of prazosin but achieved far greater potential affinity, such as STK545231 (see Table 3 and Appendix Table 1). Even prazosin with its smaller molecular weight yet relatively low potential affinity across each ortholog was able to show an IC_50_ value for human Siglec-1 using inhibition ELISA (see Table 3). This indicates that although such descriptor data can help inform, there exists limitations in this context.

It was shown that all GCUT-SMR descriptors showed negligible association to ligand affinity for the receptor site. The GCUT_SMR group of descriptors are a group of descriptors which measure polarizability of a ligand according to their atomic contribution to molar refractivity (Inc. 2008). SlogP_VSA0-9 are descriptors for the subdivided surface area based on the approximate access to van der Waal interaction within the binding site (Inc. 2008). SlogP_VSA0 indicates preference for highly polar molecules as it represents the summed contribution for all atoms where slogP is less than − 0.4. Each category from SlogP_VSA0 to SlogP_VSA9 indicates different level of logP contributions from atoms involved, so that hydrophobic ligands are progressively favoured if the descriptor indication is positive. Although the descriptor SlogP_VSA0 is heavily preferred in both mouse and rat compared to human Siglec-1, the increased hydrophobicity within the binding site of mouse Siglec-1 may still be evident (due to Leu107). Higher categories of SlogP_VSA including SlogP_VSA6 to SlogP_VSA8 show more favourable contributions for mouse Siglec-1 even if slight, in comparison to both human and rat Siglec-1. This indicates a more exposed hydrophobic surface which can be visualised when looking at the mouse Siglec-1 binding site in comparison to the other orthologs in Fig. 3. It was shown that between the variants of Siglec-1 examined, there was a moderately positive association for human Siglec-1 based on SlogP_VSA0, with mouse and rat Siglec-1 both showing a very strong positive association for this descriptor. Trends regarding the other descriptors in this class were mixed with no other strong relationship existing except for SlogP_VSA2 and its negative association for mouse Siglec-1. There were however numerous examples of moderately negative associations existing for rat and mouse Siglec-1 among the other SlogP_VSA descriptors.

Italic, negative; bold, positive.

After assessing the training set of compounds and comparison to the test set for each of the Siglec-1 variant assessed, some differences were seen between receptor N-termini (see Table 5). Comparison between the predicted affinity of compounds using RSAR analysis and molecular docking in MOE shows how negative residual values indicate that the predictive RSAR model corresponds to less affinity than molecular docking predictions, while positive residual values indicate that RSAR analysis predicted that there would be better affinity than what molecular docking suggested. Residual values indicate the degree to which a compound from the test set corresponded with the model proposed from the training set (see Appendix Table 2 for a direct comparison between predictive affinity and that obtained from molecular docking simulations). If residual values are lower, this generally suggests greater congruence with the MLRM created from the training set. This included that STK545231 had heightened residual value in both human and rat but not in mouse (see Table 5). Lutein, which was used as a decoy ligand, had a comparatively small residual value in rat in comparison to mouse and human Siglec-1. This same relationship could be seen for STK548837 which also showed a comparatively small residual for rat Siglec-1. The opposite was seen for STK882436 where mouse Siglec-1 was shown to have a relatively small residual value compared to human and rat. Other ligands seemed to strongly correspond to the linear regression models posed by the training set as shown by their relatively small residual value in each Siglec-1 variant.

**Table 5:** Comparison of predictive binding affinity and observed in silico affinity

Compound	Mouse predictive (kcal/mol)	Mouse residual (kcal/mol)	Human predictive (kcal/mol)	Human residual (kcal/mol)	Rat predictive (kcal/mol)	Rat residual (kcal/mol)
Prazosin	− 5.52	− 0.30	− 5.91	0.27	− 5.46	0.05
Tamsulosin	− 6.01	− 0.25	− 6.30	0.54	− 5.85	0.16
Methotrexate	− 5.77	− 1.02	− 7.40	0.93	− 5.54	− 1.09
STK545231	− 7.37	0.34	− 8.11	1.58	− 7.68	1.36
STK516011	− 6.72	0.78	− 6.70	0.64	− 5.73	− 1.00
STK548837	− 8.56	1.77	− 8.11	0.88	− 7.22	0.27
STK882436	− 6.79	− 0.50	− 7.55	1.43	− 7.67	1.26
GA	− 6.57	1.27	− 6.57	1.46	− 5.54	− 0.02
6MITC	− 4.96	0.12	− 5.32	0.74	− 5.01	0.40
Cortisol	− 5.02	− 0.01	− 5.42	0.43	− 5.33	0.58
Lutein	− 8.91	1.65	− 8.79	1.62	− 6.16	0.05

### Enzyme linked immunosorbent assay for Siglec-1 inhibition

**Fig. 6:**
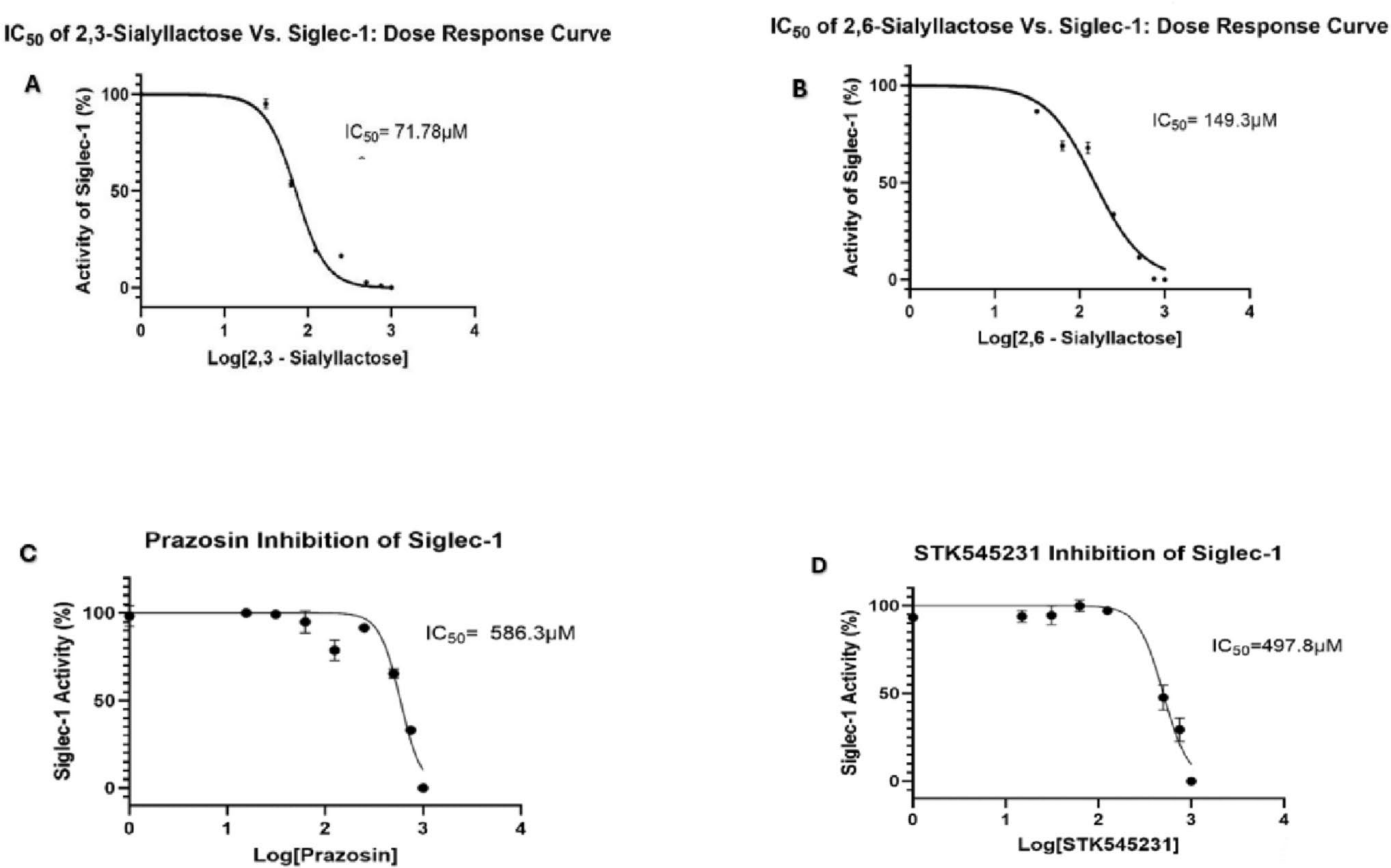
Siglec-1 inhibition ELISA for compounds which recorded IC_50_ values

The ELISA for Siglec-1 inhibition was performed on the basis of previously published protocols for Siglec inhibtion ELISA (Koliwer-Brandl et al. 2011). A standard curve was produced at the time of screening compounds, which were all completed in triplicate. After screening the selected compounds, four demonstrated sufficient inhibition to allow IC_50_ value determination (see Fig. 6) (Le Berre et al. 2022). The data points in Fig. 6. show average Siglec-1 activity (%) over three occasions in triplicate (*n* = 9) with bars indicating standard deviation at each point. The lowest IC_50_ values recorded were that of the natural ligands 2,3- and 2,6-sialyllactose at 71.78 µM (95% CI, 65.61 to 78.78) and 149.3 µM (95% CI, 123.3 to 179.3), respectively (see Fig. 6). Two additional compounds, which were non-natural ligands and previously undescribed in this context, also registered IC_50_ values. Both Prazosin and STK545231 showed similar values at 586.3µM (95% CI, 525.9 to 641.3) and 497.8 µM (95% CI, 418.9 to 557.2) respectively (see Fig. 6).

### Siglec-1 induction in HAPI cells

**Fig. 7:**
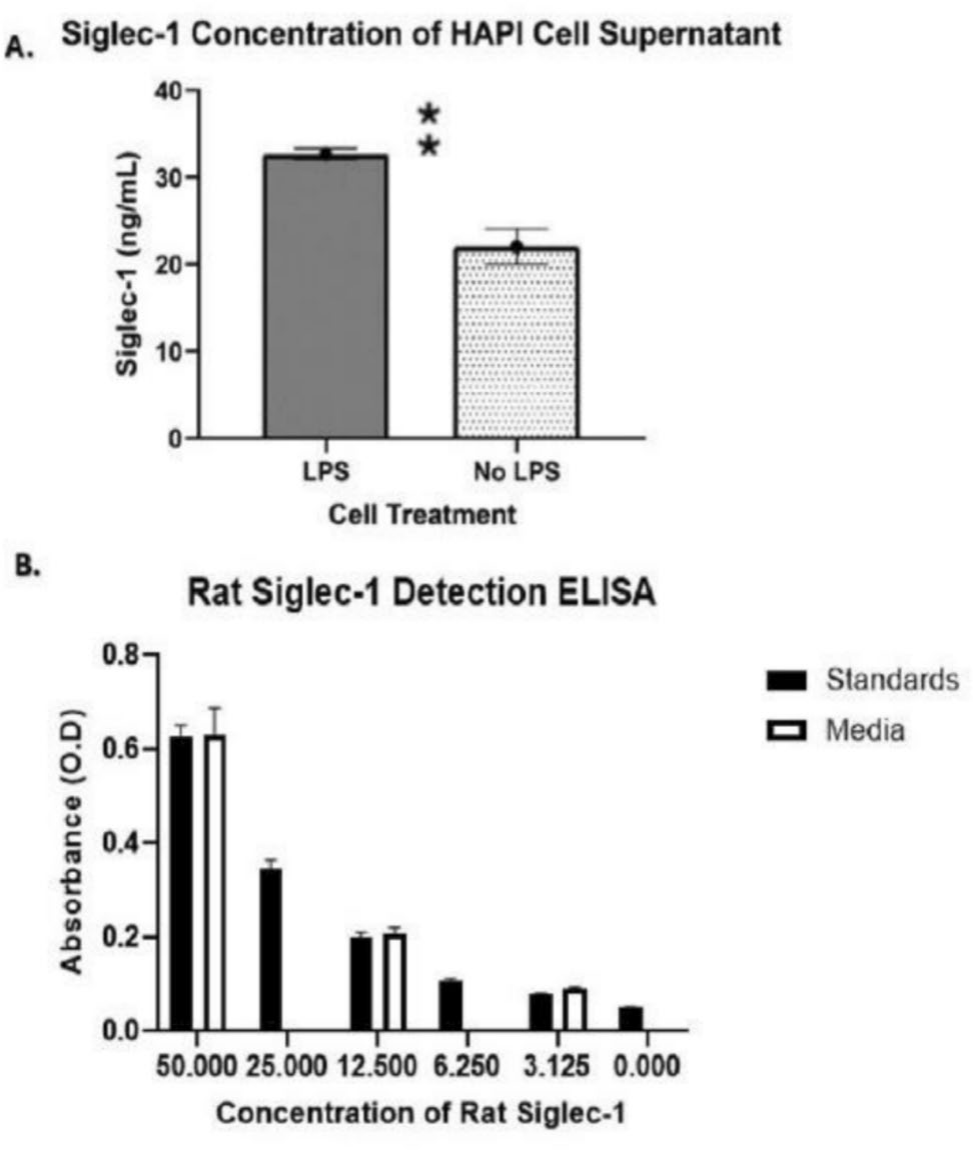
**a**, **b** The Induction of Siglec-1 after 24-h LPS exposure (**a**) and ELISA standards compared to normal cell growth media (**b**)

Siglec-1 expression was significantly induced in HAPI cells following treatment with 10 ng/mL of LPS for 24 h (see Fig. 7a). Quantification of soluble Siglec-1 (sSiglec-1) in the cell supernatant showed that LPS-treated HAPI cells shed significantly more sSiglec-1 compared to cells treated with normal growth media. The * shows significance at the indicated result, where ⁑ indicates significance of *p*-value > 0.001. It was also shown that Siglec-1 was shed within supernatant of cells treated with media only to a lesser degree. There was no significant difference between standards used in buffer and when diluted in cell media itself when analysed by Siglec-1 detection ELISA (see Fig. 7b).

### Resazurin analysis

**Fig. 8:**
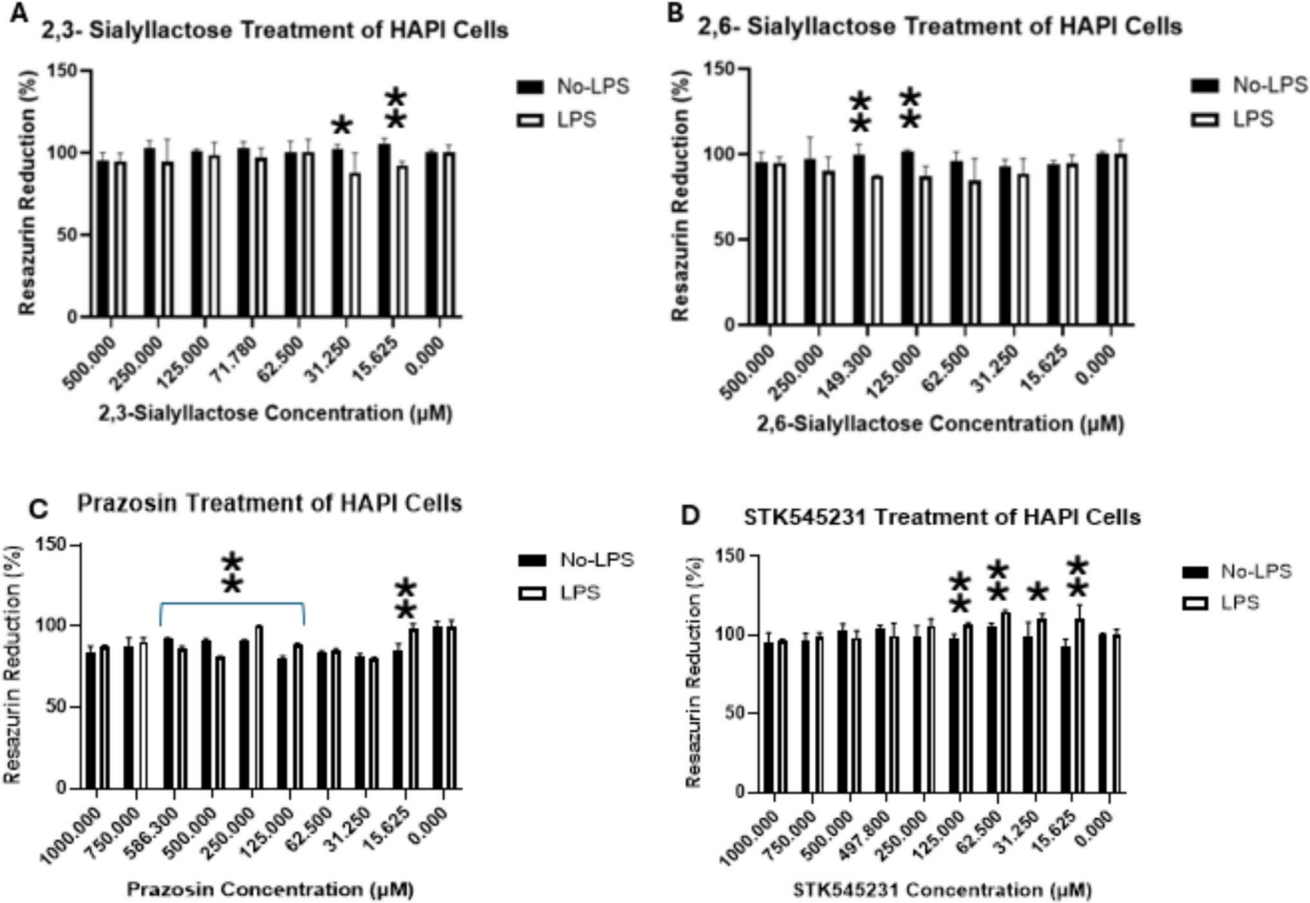
**a**–**d** The results of resazurin analysis in compounds that were able to be defined with IC_50_ values for their inhibition of human Siglec-1. * indicates significance of *p*-value > 0.01. ⁑ indicates significance of *p*-value > 0.001

**Fig. 9:**
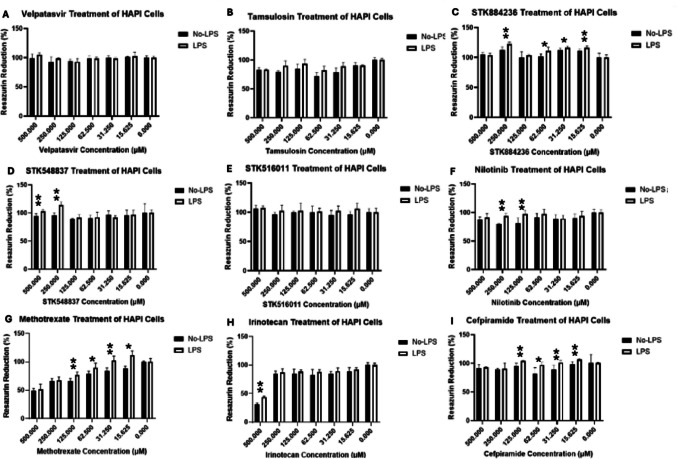
**a**–**i** The results of resazurin analysis in compounds that could not be defined with IC_50_ values for their inhibition of human Siglec-1. * indicates significance of *p*-value > 0.01. ⁑ indicates significance of *p*-value > 0.001

Resazurin analysis was conducted on all chosen compounds which included those which were selected from in silico analysis and from clinical interest (see Figs. 8 and 9). LPS stimulation was used for each compound as it was shown in this study to significantly increase Siglec-1 expression (see Fig. 7a). Normal cell growth media was used as a control for each compound. The cytotoxic effects of the tested compounds were determined by measuring resazurin reduction in HAPI cells both with and without LPS treatment (see Figs. 8 and 9). The bars of each graph in Figs. 8 and 9 represent averages of resazurin reduction on three occasions in triplicate (*n* = 9) with standard deviation. For compounds with previously recorded IC_50_ values, additional concentrations were screened to ensure the data spanned at least two points higher than the IC_50_ concentration (see Fig. 8b). In the case of 2,3-Sialyllactose and STK545231, three concentrations above the IC_50_ values were assessed due to the IC_50_ concentrations being near the value of the next closest measured concentration (see Fig. 8a, d).

Within the compounds that provided IC_50_ values for human Siglec-1 inhibition, different trends emerged depending on the compound and the concentration that the significant results were observed (see Fig. 8a–d). Both 2,3- and 2,6-sialyllactose showed significant results under No-LPS treatment conditions. At concentrations of 31.250 µM and 15.625 µM, 2,3-sialyllactose was shown to have significantly higher levels of resazurin reduction, while this was also seen for 2,6-sialyllactose at 149.3 µM and 125 µM. Prazosin indicated that there was a significant increase in resazurin reduction in the No-LPS treatment group at concentrations 586.3 µM and 500 µM (see Fig. 8c). This trend reversed when there were significant increases in resazurin reduction in the LPS treated groups for prazosin at concentrations 250 µM, 125 µM and 15.625 µM (see Fig. 8c). STK545231 showed significant results at concentrations 15.625–125 µM under the conditions of LPS treatment.

Resazurin analysis was performed up to 500µM for the remaining compounds where an IC_50_ value could not be defined by the Siglec-1 Inhibition ELISA. Toxicity was rarely an issue for most compounds in this category, even at higher concentrations. However, methotrexate and irinotecan were notable exceptions, showing substantial reductions in metabolic activity (see Fig. 9g, h). Methotrexate exhibited toxicity across every concentration tested, except at 15.625 µM, while irinotecan displayed considerable toxicity only at 500 µM (see Fig. 9). Significant results were observed for HAPI cells stimulated with LPS. These significant results were observed with Methotrexate and Cefpiramide across the 15.625–125 µM range; STK884236 at 31.250 µM, 62.5 µM, and 250 µM; STK548837 at 250 µM and 500 µM; and Nilotinib at 125 µM and 250 µM (see Fig. 9). Irinotecan displayed significant effects only at 500 µM in LPS treated cells (see Fig. 9). For tamsulosin, velpatasvir and STK516011 no significant results were found in either treatment condition at any concentration.

## Discussion

Despite Siglec-1 often being cited as a highly conserved receptor, binding site changes were observed between the mouse, human, and rat orthologs, reflecting the variation in their N-terminal domain amino acid sequences. This is most notable with the substitution of Leu107 in the mouse crystal structure to Ser107 in that of rat and human, making the binding site in human Siglec-1 resemble to a greater degree rat Siglec-1 than that of mouse. For the rest of the domain, rat and mouse Siglec-1 showed a higher degree of similarity, with their amino acid sequences deviating by only approximately 11%. In contrast, the sequence variation was significantly greater when compared to human Siglec-1, showing approximately 21% variation between rat and human, and 22% between human and mouse. Despite these substantial sequence differences, the overall structural integrity of the N-terminal domain remained largely conserved across all three mammalian species, as evidenced by the low RMSD values previously reported. Such species-specific differences must be addressed, as comparable discrepancies in other highly conserved receptors, such as Siglec-2 (CD-22), have shown functional divergence between human and murine examples. This functional gap previously necessitated the development of transgenic mice possessing human CD-22 (Huki CD-22 mice) to accurately study its effects in vivo (Wöhner et al. 2012).

Differences in the rendered lipophilic surface of Siglec-1 were observed across the orthologs when the X-ray crystal structure of Siglec-1 (1QFP) was compared to the of the homology models for human and rat. These alterations in lipophilicity patterns extended to the binding site of each ortholog. This may have theoretical ramifications for studying Siglec-1 as the molecular interactions that are formed in the binding site may be less comparable depending on the species origin. The complex achieved when the ligand BENZ was docked to murine Siglec-1 (1QFP) did however show that all critical molecular interactions were maintained as per the original X-ray crystal structure. When compared to the X-ray crystal structure of 1OD9 (murine Siglec-1 bound to BENZ), the RMSD value (1.62Å) showed that the docking procedure was successful (Abreu et al. 2012; Vittorio et al. 2024). The same docking procedure achieved a highly similar set of molecular interactions for human and rat Siglec-1, although there was evidence that the polymorphisms that exist in and near the binding site across the orthologs of Siglec-1 may have an impact on how ligands are treated when bound. Apart from the known binding site polymorphism of Leu107 (mouse) or Ser107 (human or rat), other distal polymorphisms can contribute to this effect. One such example was found to be at amino acid position 103 where each ortholog of Siglec-1 studied had a different polymorphism. These alterations although limited overall had noticeable variation with shared binding site amino acids compared to murine Siglec-1. This was despite Siglec-1 orthologs having highly similar RMSD values. Ultimately these cumulative but minor changes to Siglec-1 variants may have caused the BENZ ligand to adopt a slightly changed yet similar complex with Siglec-1 due to polymorphism across orthologs (Berndsen et al. 2016).

Molecular docking is theoretical and molecular binding affinities are not expected to correlate exactly with that of experimental means of measuring inhibition such as IC_50_ (Pantsar and Poso 2018). Comparison of binding affinity to molecular docking is therefore not possible; molecular docking serves to guide the experimental process but cannot replace it. Limitations exist in that molecular docking does not include the impact of solvent in binding interactions; other interactions may also be poorly represented in algorithms used in molecular docking (Pantsar and Poso 2018). The inherent reduction in flexibility is another consideration that impacts the realism of molecular interactions between ligand and target molecules such as the case of Siglec-1 and BENZ (Pantsar and Poso 2018). Furthermore, the use of homology models presents some uncertainty compared to the true structure of a protein (Ramsbottom et al. 2018). A more accurate way to predict non-covalent interactions in molecular docking scenarios would be to use a quantitative mechanics approach but most docking algorithms still use a more classic approach used in this paper or similar (Pantsar and Poso 2018).

Ligand affinity data from the molecular docking analysis using MOE showed some unexpected results. The results observed showed marginally higher affinity for 2,6-sialyllactose compared to 2,3-sialyllactose in human Siglec-1. In both rat and mouse Siglec-1, it was shown that 2,3- sialyllactose had greater affinity than 2,6-sialyllactose. Siglec-1 has previously been associated with higher affinity for alpha-sialosides in 2,3-linked orientations compared to 2,6- and 2,8-linked alpha-sialosides (Crocker et al. 2007; Bornhöfft et al. 2018). This was however reflected in the inhibition ELISA for human Siglec-1. The four compounds that showed inhibitory activity in vitro (by returning IC_50_ values) did not possess the highest theoretical binding affinity for human Siglec-1 in the molecular docking analysis. In the case of 2,3-sialyllactose, an IC_50_ value of 71.78 µM was obtained compared to 149.3 µM for 2,6-sialyllactose. Meanwhile, both Prazosin and STK545231 also recorded IC_50_ values from the direct Siglec-1 ELISA, of 586.3µM and 497.8µM, respectively. These results did not correlate with the corresponding affinities from molecular docking. Molecular docking analysis indicated that STK545231 reported better affinity than 2,3- and 2,6-sialyllactose, both of which had superior affinity when ELISA tested.

The utility of in silico research, which accelerates the pre-clinical process, aids in lead compound identification, and reduces drug development costs, is well-established (Aaftaab et al. 2019, Gurung et al. 2021; Adelusi et al. 2022). The results from this study’s molecular docking must be interpreted considering methodological limitations, which likely contributed to the poor correlation between predicted and ELISA-determined affinities (Adelusi et al. 2022). Specifically, the docking used in this study did not account for the effect of solvents and their interactions that influence ligand binding to protein binding sites (Adelusi et al. 2022). This exclusion is particularly relevant given the numerous hydrophilic side chains within the Siglec-1 binding site, including the critical Arg97, which forms an essential salt-bridge interaction with alpha-sialoside ligands (Adelusi et al. 2022). Furthermore, while MOE allowed for some flexibility in both the Siglec-1 side chains and ligand torsion angles to partially replicate an induced-fit interaction (Sacan et al. 2012; Adelusi et al. 2022; Ivanova and Karelson 2022), this attempts to enable a more realistic representation of the molecular motion of amino acid sidechains which can alter depending on temperature, pH, or surrounding molecular environment (Adelusi et al. 2022; Ivanova and Karelson 2022). However, the predictability of such models in comparison to true intermolecular interactions and movement still has limitations (Sacan et al. 2012). These aspects may go some way in explaining how, in the molecular docking observed, 2,6-sialyllactose had greater binding affinity than 2,3-sialyllactose. This contrasted with existing literature and even in the Siglec-1 inhibition ELISA utilised in this study, 2,3-sialyllactose is known to have superior affinity. A more rigorous in silico approach would be to now conduct molecular dynamics simulations in which solvent is explicitly incorporated and the dynamic nature of ligand binding monitored over a time course relevant to molecular interactions (Hussein et al. 2023).

The molecular docking and RSAR analysis performed across species for Siglec-1 in this study revealed that although structural similarities existed between the orthologs of Siglec-1 examined, distinct differences in the binding site were also apparent. Of particular interest was the close structural homology between the human and rat Siglec-1 N-terminal domains compared to the murine X-ray crystal structure. This similarity is highlighted by the presence of have Ser107 in human and rat, compared to Leu107 in the murine Siglec-1 N-terminal domain. This closer relationship between the human and rat binding sites suggests that results from the human Siglec-1 ELISA inhibition study may be more directly interpretable for the rat HAPI cell Resazurin analysis. However, the limited predictive power of the in silico data regarding cross-species binding may be attributed to methodological limitations. This includes the exclusion of solvent effects and although attempts at induced fit modelling during docking were pursued this is considerably static and theoretical in comparison to true molecular behaviour (Ramírez and Caballero 2016). An additional constraint was the limited size of the training set used for RSAR due to the scarce availability of known biological ligand data; constructing a larger training set would be required to obtain more detailed, reliable information about the binding site characteristics (Inc. 2008; Ul-Haq et al. 2016; Duchowicz 2018; Gurung et al. 2021; Temml and Kutil 2021; Lévêque et al. 2022; Mamada et al. 2022). Despite these limitations, the linear regression analysis from RSAR provided theoretical insight into preferred ligand features (see Table 4). Trends apparent in the descriptor analysis (see Table 4) suggested a preference for ligands that facilitate van der Waals interactions in certain regions of the binding site (SlogP_VSA0 but not others), while disfavouring ligands with a larger molecular mass and an overall negative charge.

When developing the training set for RSAR analysis, it was noted that the amount of available biological data to use was severely limited. This meant that a heavy reliance was made to the known experimental compounds that had showed inhibition activity. Another consideration was the utilisation of compounds that were known non-binders to the active site of sigles-1 and for this the Decoy1_mod from the decoy library was utilised by adding it to the training set (https://dude.docking.org/) (Huang et al. 2006; Mysinger et al. 2012; Temml and Kutil 2021). This decoy was to function as a negative control in the absence of experimental data concerning non-binding compounds for the Siglec-1 binding site (Mysinger et al. 2012). The negative control used for the training set of compounds was selected from that of a closely related structural target with a similar Greek-key topology from the DUD-e database rather than Siglec-1. A more reliable negative control would require biological data regarding known non-binding compounds for Siglec-1, which is currently lacking (Temml and Kutil 2021). Once established, the training set contained compounds comprised of natural ligands to Siglec-1 (i.e. sialic acid, 2,3- and 2,6-sialyllactose), known experimentally determined inhibitors from previous studies (i.e. BENZ, BPC-Neu5Ac and 2-Phenyl- Prop5Ac) and known compounds examined via ELISA for inhibition to human Siglec-1 (velpatasvir, cefpiramide, nilotinib and irinotecan). The test set was devised from compounds of interest and additional compounds which are structural diverse in comparison to natural ligands. This enabled binding differences to be probed amongst the three orthologs of Siglec-1. Further deciphering of additional biological data would expand the training set used in future QSAR analysis of Siglec-1, which could enable the theoretical prediction for applicable ligand design (Tukur et al. 2019). Ideally for QSAR, the test set of compounds would be approximately 20% of the volume for the total dataset with 80% of compounds in a dataset being used for the training set (Tukur et al. 2019). By having a larger training set in comparison to the test set, Tukur (2019) was able to have a more robust QSAR model enabling better forecasting of binding affinity for test set compounds (Tukur et al. 2019). The lack of available data used in this study for the two sets (training and test set) of compounds would have reduced the capacity for reliable QSAR analysis to have precision for MLRM (Tukur et al. 2019).

The HAPI cell line was selected as the microglial model due to its reported phenotypic and functional profile being the most comparable to primary microglial cells when evaluated against other immortalised lines, such as BV2, N9, HMO6, and EOC cells (Stansley et al. 2012). Despite this, it is known that the use of HAPI cells and other immortalised microglial cell lines should be interpreted with caution in directly relaying the results obtained from their use with that of primary microglial cells. Although they may have similar phenotypic profiles, the release of cytokines such as IL-1B, TNF-α, IL-6, and MCP-1 following LPS treatments has been shown not to match exactly that of primary microglial cells (Stansley et al. 2012). More study needs to be conducted to prove that HAPI cells are an appropriate substitution for human primary microglial cells when studying human Siglec-1, despite the homological similarities shown in this study. Further ELISA and in vitro analysis utilising rat Siglec-1 to assess inhibitory potential of ligands should complement a more robust in silico analysis.

Analysis of Siglec-1 shed into HAPI cell supernatant confirmed that Siglec-1 is upregulated from induction with LPS after 24 h of exposure compared to that of untreated HAPI cells (Forrester et al. 2018). This finding is consistent with the established role of microglia as innate immune cells and reflects previous studies showing upregulation of Siglec-1 during acute inflammation (Forrester et al. 2018). The increased expression of Siglec-1 in LPS treated cells can be explained by the actions of LPS on Toll-like receptor 4 (TLR4) which, when bound, increases the expression of interferon-alpha (IFN-α) resulting in upregulation of Siglec-1 (Sundar and Sires 2013; Wu et al. 2016). The level of detectable, shed, Siglec-1 found in LPS treated HAPI cells from this study was expected, considering levels of up to 276.1 ng/ml were found in blood plasma from patients with systemic lupus erythematous (SLE) (York et al. 2007; Rose et al. 2013; Oliveira et al. 2018). The finding that Siglec-1 was upregulated in untreated media suggests this is a direct effect of the media itself. Under normal conditions, the BBB isolates the central nervous system from the components of blood plasma, which would otherwise induce the expression of Siglec-1 in microglial cells (Siddiqui et al. 2019). It is reasonable to conclude that the baseline expression of Siglec-1 in untreated cells is due to the exposure to components of cell media.

Resazurin analysis was conducted in all chosen compounds, which were selected from in silico analysis and from clinical interest under conditions of normal growth media or under 24-h induction of LPS at 10 ng/mL. The Resazurin analysis revealed that most screened compounds exhibited low cytotoxicity in HAPI cells across both LPS-treated and untreated conditions over 24 h. Notable cytotoxicity was only evident at higher concentrations. For instance, irinotecan only showed apparent cytotoxicity at 500 µM. Methotrexate showed an almost linear increase in cytotoxicity as the concentration increased. The observed cytotoxicity of both methotrexate and irinotecan was anticipated, as their mechanisms of action are well-established for these known cytotoxic agents (Pavillard et al. 2002; Vezmar et al. 2003; Bailly 2019; De Lisa et al. 2020; Hamed et al. 2022). The persistent toxicity observed across most methotrexate concentrations, versus the limited effect of irinotecan (primarily at 500 µM), may be attributed to methotrexate’s multiple mechanisms of action and the limited 24-h observation period. Importantly, cytotoxicity was not a reported problem at any tested IC_50_ value (for compounds found to have a IC_50_ value during ELISA). The results suggest that Siglec-1 inhibition is unlikely to be a direct cause of cytotoxicity in HAPI cells.

The significance observed during resazurin analysis of HAPI cells when treated with compounds, showed that in most instances LPS treated cells either had possible proliferation or improved survivability. However, the results would suggest that this was mainly seen in compounds that were not able to achieve inhibition of Siglec-1 during the human Siglec-1 inhibition ELISA. In the four compounds that were able to have a definable IC_50_ values, significant results obtained at IC_50_ or higher were likely to see higher resazurin reduction or metabolic activity when cells were not treated with LPS. In the same IC_50_ compounds, significant results discovered at concentrations lower than the IC_50_ value indicated that the reverse relationship may be occurring where again LPS treated cells had higher levels of metabolic activity. Further research is required to elucidate whether there is a possible mechanism regarding Siglec-1 inhibition that could be instigating a trend in this instance. There may be multiple explanations for the observations seen, firstly LPS may induce proliferation among microglial cells, as has been shown previously (He et al. 2021). It is also known that LPS induces morphological changes in microglial cells, where cells adopt a more active and dynamic state which at times can seem ameboid like in appearance (Kim et al. 2024). Although morphological changes were not assessed in this study, such morphological changes are a common feature of immune activation of microglial cells (Gao et al. 2023; Kim et al. 2024). Supporting this, LPS induction has been shown to positively regulate genes associated with proliferation whilst also suppressing genes that negatively regulate the cell cycle (He et al. 2021). This gene expression profile was observed in HAPI cells pre-treated with LPS (at 10 ng/mL and 100 ng/mL) and those treated with poly(I:C) (at 3 µg/mL and 10 µg/mL), in comparison to controls (He et al. 2021). Additionally, it was previously demonstrated that moderately dosed LPS can extend the lifespan of cultured primary microglial cells by suppressing autophagy and apoptosis (Kaneko et al. 2009). This could account for the resistance to cytotoxicity and perhaps, the proliferation of HAPI cells observed at various levels of compound exposure within the LPS treatment group.

Siglec-1 has been previously hypothesised to play a crucial role in immune suppression by inhibiting the NFkB pathway, which in turn can suppress TNFα expression (Wu et al. 2016). This mechanism was initially proposed in the context of sepsis, where Siglec-1, acting via SyK intracellular signalling, leads to an increase in TGF-β1. The increase in TGF-β1 in turn suppresses NFkB, thereby inhibiting the formation of TNFα which would otherwise be upregulated through the actions of LPS acting on its receptor, TLR4 (Wu et al. 2016). This mechanism has since been alluded to in other Siglec-1-expressing cells. For instance, in human synovial mesenchymal stem cells (hSMSCs), the blockade of Siglec-1 using siRNA reduced signalling through the TGF-β1/SMAD pathway (Chen et al. 2024). Considering these previous findings, further study is warranted to fully elucidate the mechanistic actions of Siglec-1 regarding the inflammatory effects on microglia.

## Conclusion

The differences between Siglec-1 binding sites across mammalian species could potentially have an impact on studies, as polymorphic changes might alter their functionality. For example, murine Siglec-1 was found to have Leu107 in its binding site, while both rat and human shared Ser107. Notably, this occurred despite the overall structural convergence among all three variants considered in this study. RSAR analysis although limited in scope showed that there was the potential for differences in human and rat Siglec-1 to cause differences in ligand affinity, even with the shared binding site polymorphism. The results from in silico analysis are not to be interpreted without some awareness of their limitations. For instance, it is well-established that 2,3-linked alpha sialosides exhibit a stronger affinity for Siglec-1 compared to 2,6-linked alpha sialosides. The results from this study confirm findings in existing literature, which previously recorded that 2,3-sialyllactose has a higher affinity for Siglec-1 than 2,6-sialyllactose. The same result was seen in the Siglec-1 inhibition ELISA used in this study, but in silico analysis showed a slight reversal of this relationship. Current limitations which exist for in silico analysis, notably the absence of water or solvent from the simulated binding interactions and the overall static behaviour of the Siglec-1 orthologs, could help in explaining these observations. Further study is required in this area, with a larger pool of in vitro data to build a more robust training set for QSAR analysis to better elucidate differences in Siglec-1 variants. Expression of Siglec-1 in HAPI cells was confirmed using ELISA and that the expression can be upregulated under LPS stimulation, highlighting the successful use of HAPI cells as a neurology model in the study of Siglec-1. While additional analysis of binding affinities against human Siglec-1 should be conducted, the in silico results here suggest that rat Siglec-1 may serve as a more comparable model than murine Siglec-1, given their polymorphic similarities. This should be considered in context with the known limitations of HAPI cells as an immortalised microglial model. Additionally, comparison of any in silico data against that of ELISA inhibition analysis utilising rat Siglec-1 for comparison to other Siglec-1 variants would be necessary. Based on the resazurin assay results, there is a strong suggestion that LPS stimulation could induce a proliferative effect on microglial cells. These findings also suggest that Siglec-1 inhibition is unlikely to be toxic to HAPI cells in vitro.

## Supplementary information

Below is the link to the electronic supplementary material.

ESM 1 (DOCX 294 KB)

## Data Availability

All source data for this work (or generated in this study) are available upon reasonable request.

## Declarations

The authors declare that no AI tool has been used for the generation of text or figures.
