# Supplementary material for: In silico and in vitro investigations of novel Siglec-1 inhibitors in a microglial cell model
Source: Naunyn Schmiedebergs Arch Pharmacol. 2026 Jan 21;399(6):9203–27. doi: 10.1007/s00210-025-04962-7 (PMC13086771; doi:10.1007/s00210-025-04962-7)
Supplement: Supplementary file 1 — (DOCX 294 KB) [file 210_2025_4962_MOESM1_ESM.docx]

**Supporting Materials**

**APPENDIX

Table 1. Compounds Utilised in RSAR**

| **Potential Ligands** | **Ligand Structure** |
| --- | --- |
| ***Training Set Compounds*** |  |
| 2-Phenyl- Prop5Ac  Formula: C_19_H_27_NO_9_  FW: 413.4 g/mol | 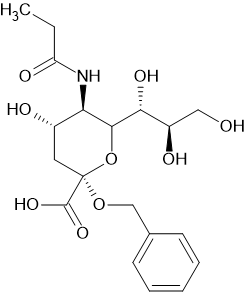  SMILES:CCC(=O)N[C@@H]1[C@H](C[C@@](O[C@H]1[C@@H]([C@@H](CO)O)O)(C(=O)O)OCC2=CC=CC=C2)O |
| Me- α -9-N-(biphenyl-4-carbonyl)-amino-9-deoxy-Neu5Ac (i.e. BPC-Neu5Ac)  Formula: C_25_H_30_N_2_O_9_  FW: 502.5 g/mol | 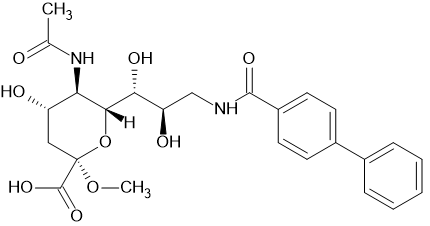  SMILES:CC(=O)N[C@@H]1[C@H](C[C@@](O[C@H]1[C@@H]([C@@H](CNC(=O)C2=CC=C(C=C2)C3=CC=CC=C3)O)O)(C(=O)O)OC)O |
| Me-α-9-*N*-benzoyl-amino-9-deoxy-Neu5Ac (i.e. BENZ)  Formula: [C_19_H_26_N_2_O_9_](https://pubchem.ncbi.nlm.nih.gov/#query=C19H26N2O9)  FW: 426.4 g/mol | 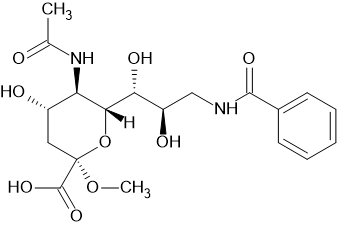  SMILES:CC(=O)N[C@@H]1[C@H](C[C@@](O[C@H]1[C@@H]([C@@H](CNC(=O)C2=CC=CC=C2)O)O)(C(=O)O)OC)O |
| Velpatasvir  Formula: [C_49_H_54_N_8_O_8_](https://pubchem.ncbi.nlm.nih.gov/#query=C49H54N8O8)  FW: 883.0 g/mol | 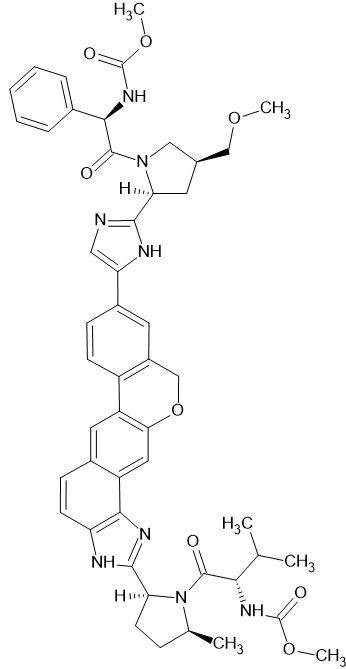  SMILES:C[C@H]1CC[C@H](N1C(=O)[C@H](C(C)C)NC(=O)OC)C2=NC3=C(N2)C=CC4=CC5=C(C=C43)OCC6=C5C=CC(=C6)C7=CN=C(N7)[C@@H]8C[C@@H](CN8C(=O)[C@@H](C9=CC=CC=C9)NC(=O)OC)COC |
| 2,6-sialyllactose  Formula: [C_23_H_39_NO_19_](https://pubchem.ncbi.nlm.nih.gov/#query=C23H39NO19)  FW: 633.6 g/mol | 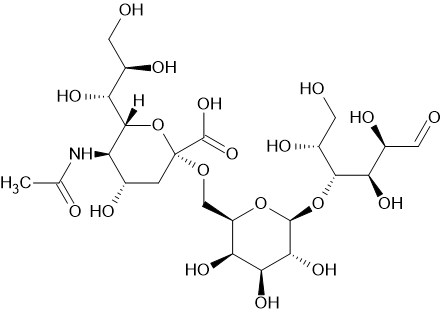  SMILES:CC(=O)N[C@@H]1[C@H](C[C@@](O[C@H]1[C@@H]([C@@H](CO)O)O)(C(=O)O)OC[C@@H]2[C@@H]([C@@H]([C@H]([C@@H](O2)O[C@H]([C@@H](CO)O)[C@@H]([C@H](C=O)O)O)O)O)O)O |
| 2,3-sialyllactose  Formula: [C_23_H_39_NO_19_](https://pubchem.ncbi.nlm.nih.gov/#query=C23H39NO19)  FW: 633.6 g/mol | 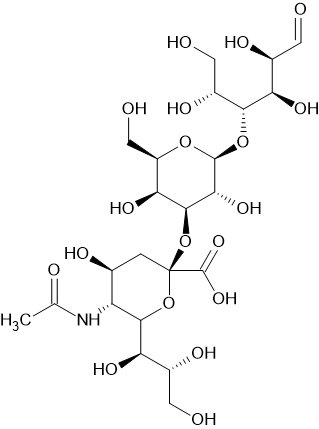  SMILES:CC(=O)N[C@@H]1[C@H](C[C@@](O[C@H]1[C@@H]([C@@H](CO)O)O)(C(=O)O)O[C@H]2[C@H]([C@H](O[C@H]([C@@H]2O)O[C@H]([C@@H](CO)O)[C@@H]([C@H](C=O)O)O)CO)O)O |
| Cefpiramide  Formula: [C_25_H_24_N_8_O_7_S_2_](https://pubchem.ncbi.nlm.nih.gov/#query=C25H24N8O7S2)  FW: 612.6 g/mol | 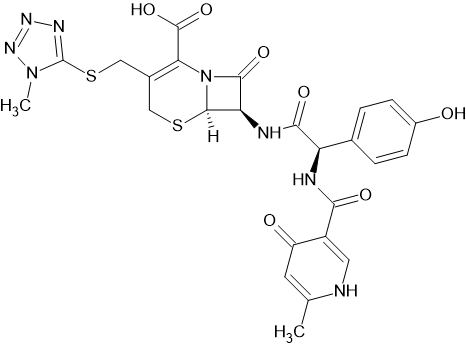  SMILES:CC1=CC(=O)C(=CN1)C(=O)N[C@H](C2=CC=C(C=C2)O)C(=O)N[C@H]3[C@@H]4N(C3=O)C(=C(CS4)CSC5=NN=NN5C)C(=O)O |
| Nilotinib  Formula: [C_28_H_22_F_3_N_7_O](https://pubchem.ncbi.nlm.nih.gov/#query=C28H22F3N7O)    FW: 529.5 g/mol | 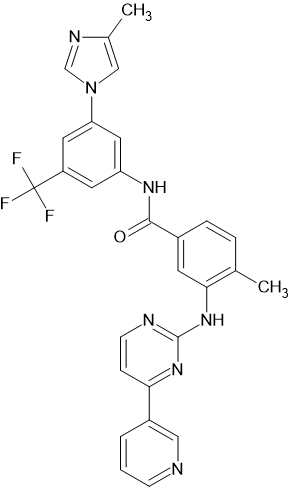  SMILES:CC1=C(C=C(C=C1)C(=O)NC2=CC(=CC(=C2)C(F)(F)F)N3C=C(N=C3)C)NC4=NC=CC(=N4)C5=CN=CC=C5 |
| Irinotecan  Formula: C_33_H_38_N_4_O_6_   FW: 586.7 g/mol | 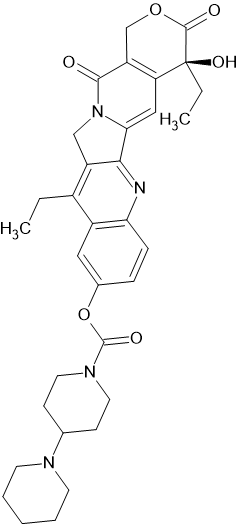  SMILES:CCC1=C2CN3C(=CC4=C(C3=O)COC(=O)[C@@]4(CC)O)C2=NC5=C1C=C(C=C5)OC(=O)N6CCC(CC6)N7CCCCC7 |
| Decoy1_mod (negative control)   Formula: C_16_H_22_N_3_O_6_^-^  FW: 352.4g/mol | 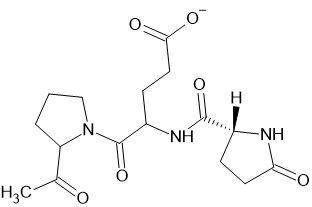  SMILES: Not available, see the DUD-e database (http://dude.docking.org) (Mysinger, Carchia et al. 2012). |
| Sialic acid   Formula: [C_11_H_19_NO_9_](https://pubchem.ncbi.nlm.nih.gov/#query=C11H19NO9)  FW: 309.3 g/mol | 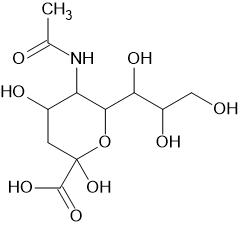  SMILES:CC(=O)N[C@@H]1[C@H](CC(O[C@H]1[C@@H]([C@@H](CO)O)O)(C(=O)O)O)O |
| ***Test Set Compounds*** |  |
| Prazosin  Formula: [C_19_H_21_N_5_O_4_](https://pubchem.ncbi.nlm.nih.gov/#query=C19H21N5O4)  FW: 383.4 g/mol | 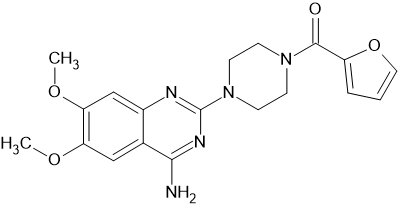  SMILES:COC1=C(C=C2C(=C1)C(=NC(=N2)N3CCN(CC3)C(=O)C4=CC=CO4)N)OC |
| Tamsulosin  Formula: C_20_H_28_N_2_O_5_S   FW: 408.5 g/mol | 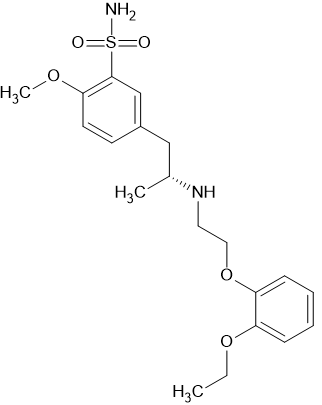  SMILES:CCOC1=CC=CC=C1OCCN[C@H](C)CC2=CC(=C(C=C2)OC)S(=O)(=O)N |
| Methotrexate  Formula: [C_20_H_22_N_8_O_5_](https://pubchem.ncbi.nlm.nih.gov/#query=C20H22N8O5)  FW: 454.4 g/mol | 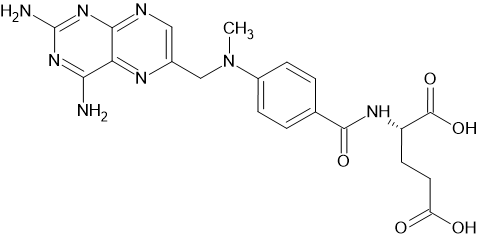  SMILES:CN(CC1=CN=C2C(=N1)C(=NC(=N2)N)N)C3=CC=C(C=C3)C(=O)N[C@@H](CCC(=O)O)C(=O)O |
| STK545231  Formula:  C_33_H_30_Cl_2_N_4_O_5_S_2_  FW:  697.7 g/mol | 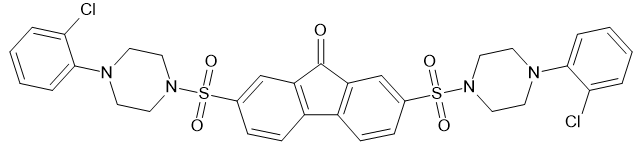  SMILES:C1CN(CCN1C2=CC=CC=C2Cl)S(=O)(=O)C3=CC4=C(C=C3)C5=C(C4=O)C=C(C=C5)S(=O)(=O)N6CCN(CC6)C7=CC=CC=C7Cl |
| STK516011  Formula: [C_28_H_32_N_2_O_7_](https://pubchem.ncbi.nlm.nih.gov/#query=C28H32N2O7)  FW: 508.6 g/mol | 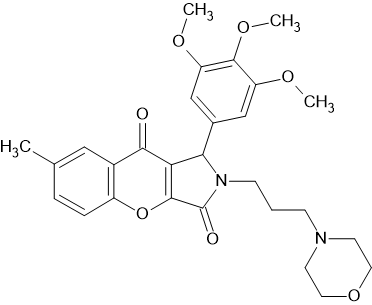  SMILES:CC1=CC2=C(C=C1)OC3=C(C2=O)C(N(C3=O)CCCN4CCOCC4)C5=CC(=C(C(=C5)OC)OC)OC |
| STK548837  Formula: [C_35_H_32_N_6_O_6_S_2_](https://pubchem.ncbi.nlm.nih.gov/#query=C35H32N6O6S2)  FW: 696.8 g/mol | 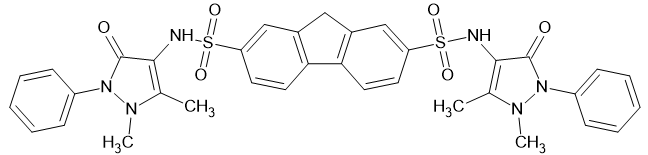  SMILES:CC1=C(C(=O)N(N1C)C2=CC=CC=C2)NS(=O)(=O)C3=CC4=C(C=C3)C5=C(C4)C=C(C=C5)S(=O)(=O)NC6=C(N(N(C6=O)C7=CC=CC=C7)C)C |
| STK882436  Formula: C_30_H_36_N_4_O_7_S  FW: 596.7 g/mol | 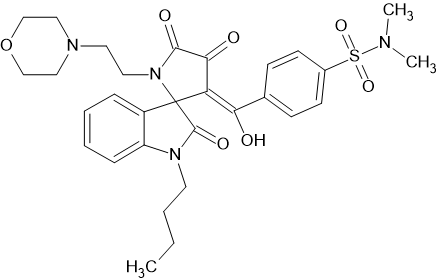  SMILES:CCCCN1C2=CC=CC=C2C3(C1=O)/C(=C(/C4=CC=C(C=C4)S(=O)(=O)N(C)C)\O)/C(=O)C(=O)N3CCN5CCOCC5 |
| Glycyrrhetinic Acid  Formula: [C_30_H_46_O_4_](https://pubchem.ncbi.nlm.nih.gov/#query=C30H46O4)  FW: 470.7 g/mol | 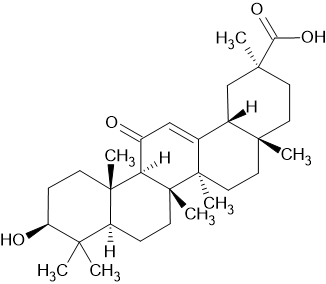  SMILES:C[C@]12CC[C@](C[C@H]1C3=CC(=O)[C@@H]4[C@]5(CC[C@@H](C([C@@H]5CC[C@]4([C@@]3(CC2)C)C)(C)C)O)C)(C)C(=O)O |
| 6-(methylsulfinyl)hexyl isothiocyanate  Formula: [C_8_H_15_NOS_2_](https://pubchem.ncbi.nlm.nih.gov/#query=C8H15NOS2)  FW: 205.3 g/mol | 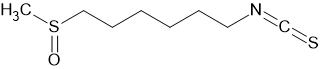  SMILES: CS(=O)CCCCCCN=C=S |
| Cortisol ^Ѱ^ Formula: [C_21_H_30_O_5_](https://pubchem.ncbi.nlm.nih.gov/#query=C21H30O5)  FW: 362.5 g/mol | 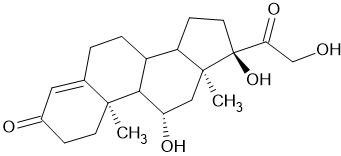  SMILES:C[C@]12CCC(=O)C=C1CC[C@@H]3[C@@H]2[C@H](C[C@]4([C@H]3CC[C@@]4(C(=O)CO)O)C)O |
| Lutein*  Formula: C_40_H_56_O_2_  FW: 568.9 g/mol | 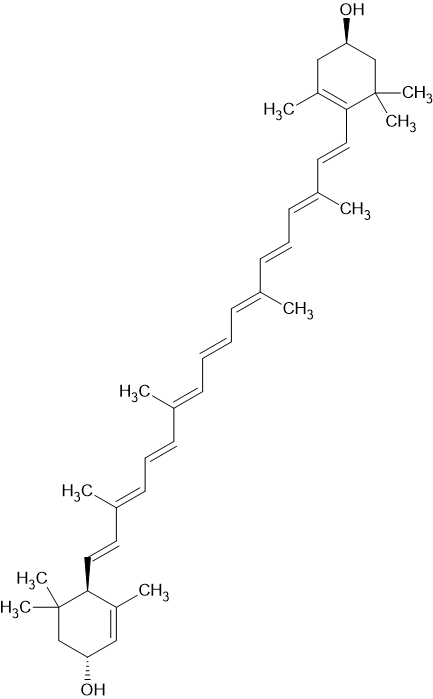  SMILES:CC1=C(C(C[C@@H](C1)O)(C)C)/C=C/C(=C/C=C/C(=C/C=C/C=C(\C)/C=C/C=C(\C)/C=C/[C@H]2C(=C[C@@H](CC2(C)C)O)C)/C)/C |

This table details the structural properties of all compounds utilised in RSAR analysis for this study.

**Table 2. Predictive Biological Activity Obtained from RSAR (Predictive) Vs. Molecular Docking Analysis**

| **Compound** | **Mouse Predictive (kcal/mol)** | **Mouse Molecular Docking Analysis (kcal/mol)** | **Human Predictive (kcal/mol)** | **Human Molecular Docking Analysis (kcal/mol)** | **Rat Predictive (kcal/mol)** | **Rat Molecular Docking Analysis (kcal/mol)** |
| --- | --- | --- | --- | --- | --- | --- |
| Prazosin | -5.52 | -5.82 | -5.91 | -5.64 | -5.46 | -5.41 |
| Tamsulosin | -6.01 | -6.26 | -6.30 | -5.76 | -5.85 | -5.69 |
| Methotrexate | -5.77 | -6.79 | -7.40 | -6.47 | -5.54 | -6.63 |
| STK545231 | -7.37 | -7.03 | -8.11 | -6.53 | -7.68 | -6.31 |
| STK516011 | -6.72 | -5.94 | -6.70 | -6.06 | -5.73 | -6.73 |
| STK548837 | -8.56 | -6.79 | -8.11 | -7.23 | -7.22 | -6.95 |
| STK882436 | -6.79 | -7.29 | -7.55 | -6.12 | -7.67 | -6.41 |
| GA | -6.57 | -5.30 | -6.57 | -5.11 | -5.54 | -5.56 |
| 6MITC | -4.96 | -4.84 | -5.32 | -4.58 | -5.01 | -4.61 |
| Cortisol | -5.02 | -5.03 | -5.42 | -4.99 | -5.33 | -4.75 |
| Lutein | -8.91 | -7.26 | -8.79 | -7.17 | -6.16 | -6.11 |

This table details both the docking scores obtained from molecular docking analysis and the predictive scores that were obtained from MOE after conducting RSAR analysis. This directly relates to the residual scores detailed earlier (see Table 5).
